# Diagnosis and Treatment of Male Infertility-Related Fertilization Failure

**DOI:** 10.3390/jcm9123899

**Published:** 2020-12-01

**Authors:** Arantxa Cardona Barberán, Annekatrien Boel, Frauke Vanden Meerschaut, Dominic Stoop, Björn Heindryckx

**Affiliations:** Ghent-Fertility and Stem Cell Team (G-FaST), Department for Reproductive Medicine, Ghent University Hospital, Corneel Heymanslaan 10, 9000 Ghent, Belgium; arantxa.cardonabarberan@UGent.be (A.C.B.); Annekatrien.Boel@UGent.be (A.B.); Frauke.VandenMeerschaut@uzgent.be (F.V.M.); Dominic.Stoop@uzgent.be (D.S.)

**Keywords:** male infertility, fertilization failure, ICSI, oocyte activation deficiencies, phospholipase C zeta (PLCζ), PLCZ1 mutations, MOAT, MOCA, HOCA, AOA

## Abstract

Infertility affects approximately 15% of reproductive-aged couples worldwide, of which up to 30% of the cases are caused by male factors alone. The origin of male infertility is mostly attributed to sperm abnormalities, of which many are caused by genetic defects. The development of intracytoplasmic sperm injection (ICSI) has helped to circumvent most male infertility conditions. However, there is still a challenging group of infertile males whose sperm, although having normal sperm parameters, are unable to activate the oocyte, even after ICSI treatment. While ICSI generally allows fertilization rates of 70 to 80%, total fertilization failure (FF) still occurs in 1 to 3% of ICSI cycles. Phospholipase C zeta (PLCζ) has been demonstrated to be a critical sperm oocyte activating factor (SOAF) and the absence, reduced, or altered forms of PLCζ have been shown to cause male infertility-related FF. The purpose of this review is to (i) summarize the current knowledge on PLCζ as the critical sperm factor for successful fertilization, as well as to discuss the existence of alternative sperm-induced oocyte activation mechanisms, (ii) describe the diagnostic tests available to determine the cause of FF, and (iii) summarize the beneficial effect of assisted oocyte activation (AOA) to overcome FF.

## 1. Introduction

Infertility is a worldwide health problem defined by the World Health Organization as the inability of a couple to achieve pregnancy after one year of regular, unprotected intercourses [1]. It is estimated to affect around 15% couples worldwide, with a higher prevalence in certain regions [2,3,4]. Infertility is attributed to male factors alone in 20 to 30% of the cases [3,4,5,6], with an overall contribution of up to 50% [2,3,5]. Male infertility is mainly caused by urogenital abnormalities, endocrine disturbances, and immunological and genetic defects [2,5,6,7,8,9], the latter being of much concern. Genetic causes of male infertility include chromosomal abnormalities, as well as single gene mutations and result in a great variety of sperm abnormalities. Common genetic defects involve microdeletions in the azoospermia factor (AZF) region on the Y chromosome [10]. Deletion of USP9Y or DAZ genes, present in the AZF region, causes azoospermia (the complete absence of sperm in ejaculates) and oligozoospermia (sperm concentration < 15 million/mL) [11,12,13]. Single gene mutations in AKAP3 and AKAP4 lead to dysplasia of the fibrous sheath and asthenozoospermia (reduced sperm motility) [14], while mutations in KLHL10 have been linked to oligozoospermia [15] and mutations in CATSPER1 cause oligo-astheno-teratozoospermia (reduced sperm concentration, motility, and abnormal morphology) [16]. Further examples include mutations in SPATA16 and DPY19L2 causing globozoospermia (a form of teratozoospermia characterized by round-headed spermatozoa lacking the acrosome) [17,18] and the gene causing cystic fibrosis, CFTR [19], whose defect is correlated to congenital bilateral absence of the vas deferens (CBAVD), a form of obstructive azoospermia.

The establishment of assisted reproduction techniques (ART), especially intracytoplasmic sperm injection (ICSI), has helped many males with suboptimal sperm parameters to conceive [20]. ICSI consists of the injection of a single sperm into the oocyte cytoplasm and allows fertilization without the need of sperm capacitation, acrosome reaction, and gamete binding and fusion [21,22]. Although the technique was first developed to treat severe male infertility and repetitive failed in vitro fertilization (IVF) cycles, it is now commonly used as the standard ART [21,22,23]. Fertilization rates obtained after ICSI treatment are reported to be between 70 and 80%, representing the most efficient ART [21,22,24]. Yet, total fertilization failure (FF) still occurs in 1 to 3% of ICSI cycles [25,26,27,28,29] and leaves these patients with few options to achieve genetically related offspring. FF following ICSI has mainly been attributed to oocyte activation deficiencies (OADs) that can be caused by both sperm- or oocyte-related factors [4,9,30].

Female infertility-related FF has been attributed to defects in proteins involved in the fertilization process (e.g., WEE2), cytoplasmic immaturity and spindle abnormalities, as well as to a low number of mature oocytes available for ICSI [4,31,32,33]. Male infertility-related FF, on the other hand, has been attributed to failed sperm head decondensation, premature chromatin condensation, sperm aster defects, and poor sperm parameters such as sperm immobility and low sperm counts [34,35,36,37]. However, the most common cause is currently believed to be a phospholipase C zeta (PLCζ) deficiency [4,9]. PLCζ is the sperm-borne oocyte activation factor (SOAF) required for triggering the necessary calcium (Ca^2+^) oscillations that cause successful mammalian fertilization [9,38]. Disruption of PLCζ by genetic mutations or reduced protein expression has been reported in patients suffering from FF after ICSI, irrespective of the sperm quality. PLCζ deficiency has been observed in normozoospermic males [39,40,41] but also in patients with abnormal sperm parameters [42,43], with globozoospermia being the most characteristic example [9,44,45]. The current evidence undoubtedly supports the role of PLCζ as a protein indispensable for successful fertilization, but there are still some unknowns that remain to be solved [46,47].

To provide the most optimal treatment options for patients suffering from FF after ICSI, it is of utmost importance to determine its origin by appropriate diagnostic tests. Heterologous and homologous ICSI models have been used to diagnose the human sperm activation potential and Ca^2+^ oscillatory pattern [48,49,50]. More recently, the evaluation of compromised PLCζ has emerged as a more simplified test that could be more easily introduced in the IVF clinics [40,51]. These diagnostic options allow the clinicians to select the most adequate treatment for the next cycle. When the OAD is sperm-related, ICSI in combination with assisted oocyte activation (AOA) has been proven to be very beneficial [52]. AOA consists of the artificial induction of Ca^2+^ oscillations in the oocyte and can be achieved by different strategies. However, the ideal AOA protocol and the use of particular artificial activation agents are still a matter of debate [24,53]. In contrast, when the OAD is oocyte-related, AOA may sometimes fail, and subsequent oocyte donation is required [50,54]. This review focuses on male factor-related FF and summarizes the current knowledge on the testis-specific PLCζ protein, as well as describes the latest advances on diagnostic tests and treatments available for the clinical management of this rare but challenging infertility condition.

## 2. PLCζ is the Primary Sperm Oocyte Activating Agent during ICSI but Alternative Factors May Contribute to Fertilization

**Figure 1:**
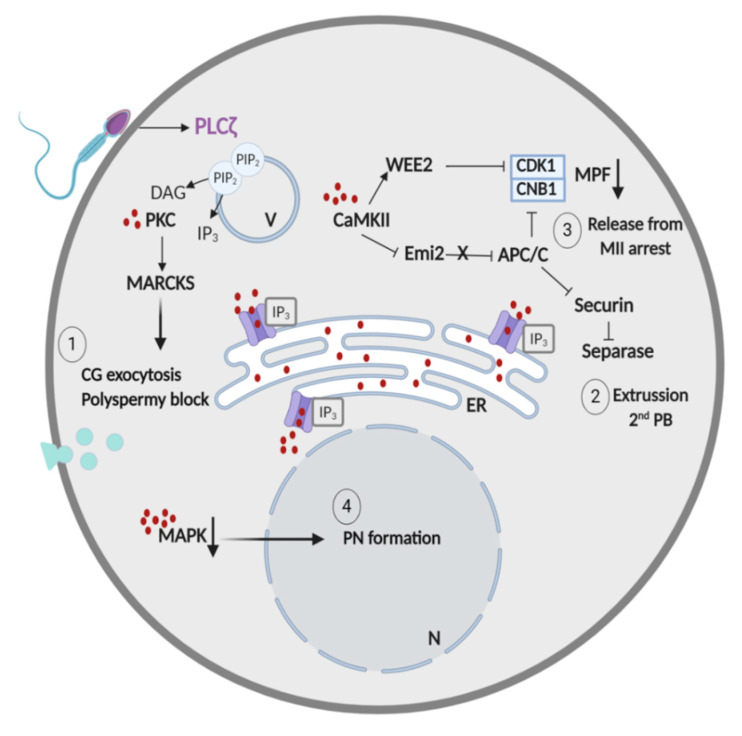
Oocyte activation pathway induced by the sperm factor PLCζ. The release of sperm PLCζ into the oocyte induces IP_3_ production, which subsequently binds to its receptor (IP_3_R) and provokes Ca^2+^ release from the endoplasmic reticulum. The Ca^2+^ oscillations will activate different oocyte kinases in a time-dependent order, allowing cortical granule exocytosis though PKC activation, extrusion of the second polar body and meiotic resumption via CaMKII activation and formation of pronuclei by MAPK inactivation. PLCζ: phospholipase C zeta; DAG: diacylglycerol; PIP2: phosphatidylinositol 4,5-biphosphate; IP_3_: inositol 1,4,5-triphosphate; PKC: protein kinase C; MARCKS: myristoylated alanine-rich C-kinase substrate; CaMKII: Ca^2+^/calmodulin-dependent protein kinase II; WEE2: Wee1-like protein kinase 2; CDK1: cyclin-dependent kinase 1; CNB1: cyclin B; MPF: maturation-promoting factor; Emi2: early mitotic inhibitor 2; APC/C: anaphase-promoting complex/cyclosome; MAPK: Mos/mitogen-activated protein kinase; V: vesicle; N: nucleus; ER: endoplasmic reticulum; CG: cortical granule; PB: polar body; PN: pronuclei; MII: metaphase II.

Normal fertilization occurs after the fusion of the male and female gametes through the process known as “oocyte activation” (OA). It is widely accepted that OA is produced when the sperm releases PLCζ into the oocyte cytoplasm [4,9,38]. PLCζ targets cytoplasmic vesicles containing phosphatidylinositol 4,5-biphosphate (PIP2) and promotes the production of inositol 1,4,5-triphosphate (IP_3_) and diacylglycerol (DAG) [55]. Then, IP_3_ molecules will bind to its receptor (IP_3_R) present on the endoplasmic reticulum (ER), which will lead to the release of Ca^2+^ from the ER stores (Figure 1). The support of long-lasting intermittent Ca^2+^ oscillations occurs due to a dual regulation of IP_3_R. Decreased intracellular Ca^2+^ concentrations increase IP_3_R sensitivity, which results in Ca^2+^ release from the ER, while elevated Ca^2+^ concentrations inhibit IP_3_R channels and halt Ca^2+^ delivery to the cytoplasm [56,57]. The Ca^2+^ oscillations will activate various oocyte kinases that will evoke different downstream events necessary for fertilization in a time-dependent order [58]. First, DAG and the released Ca^2+^ will promote the activation of protein kinase C (PKC), which will phosphorylate myristoylated alanine-rich C-kinase substrate (MARCKS) proteins, which are responsible of the induction of cortical granule exocytosis and blockage to polyspermy (Figure 1) [59]. Secondly, increased intracellular levels of Ca^2+^ will activate the Ca^2+^/calmodulin-dependent protein kinase II (CaMKII), which will in turn phosphorylate the early mitotic inhibitor 2 (Emi2) (Figure 1). Then, Emi2 will be degraded by an ubiquitin–ligase complex and will no longer be able to inhibit the anaphase-promoting complex/cyclosome (APC/C) [60,61]. Active APC/C will promote the extrusion of the second polar body by the degeneration of securin and subsequent separase inhibition [62] and the release of the oocyte from the second meiotic division by the degradation of the maturation-promoting factor (MPF) [63]. The MPF is formed by cyclin B (CNB1) and cyclin-dependent kinase 1 (CDK1). Specifically, APC/C targets the degeneration of CNB1. However, very recently, it has been reported that in order to decrease MPF levels and allow the cell cycle resumption, both the degeneration of CNB1 by APC/C and the inhibition of CDK1 by Wee1-like protein kinase 2 (WEE2), another oocyte kinase [33,64], are necessary. Finally, the Ca^2+^ oscillations will inactivate the Mos/mitogen-activated protein kinase (MAPK) pathway, which will allow the formation of pronuclei [61]. Therefore, deficiencies in any of the sperm or oocyte proteins involved in OA will most likely lead to fertilization failure.

Thus, calcium oscillations are the central landmark of fertilization and they have been reported in all mammalian species studied to date. However, the exact Ca^2+^ pattern is species-specific, with differences being found in the amplitude, duration, and frequency of the Ca^2+^ spikes [57,65]. The characteristic pattern of Ca^2+^ oscillations following ICSI of in vivo matured metaphase II (MII) human oocytes consists of a series of sharp increases in Ca^2+^ concentration followed by a return to baseline concentrations [66]. In human, the first Ca^2+^ transient is documented to appear between 30 and 90 min following ICSI and has the highest peak and longest duration. The subsequent Ca^2+^ oscillations show lower amplitude and shorter duration. On average, 2.4 Ca^2+^ transients/hour are detected during a period of 4 to 5 h [66]. It is still not known how important this specific Ca^2+^ pattern is for the fertilization and embryonic development. It has been reported that when the Ca^2+^ released reaches a certain threshold (independent of the specific Ca^2+^ profile), both oocyte activation and embryo development can occur normally [67]. Indeed, an artificial induction of few Ca^2+^ transients by different AOA protocols (see Section 4) have been reported to allow normal fertilization rates in human and establish pregnancies [24,52]. However, other studies have shown that altered Ca^2+^ patterns do not only affect the fertilization process but also have long-term effects on both the pre- and post-implantation development, especially in animal models [68,69,70]. Either way, a critical amount of Ca^2+^ released is likely to be necessary to allow the completion of the oocyte activation process [58,67]. This is evidenced by aberrant or absent Ca^2+^ oscillations when sperm from patients with fertilization failure problems is injected into mouse and human oocytes [49,50,71].

For many years, researchers have aimed to identify the main SOAF. Different candidates were proposed, among them, citrate synthase [72], a truncated form of the c-kit tyrosine kinase receptor (tr-kit) [73] and the post-acrosomal sheath WW domain-binding protein (PAWP) [74]. None of these proteins were reported to cause physiological Ca^2+^ oscillations after the fertilization of human oocytes, except for PAWP. One group reported that the injection of PAWP complementary RNA (cRNA) into mouse and human oocytes elicited Ca^2+^ oscillations [75] and even more, that high PAWP expression in sperm was correlated to higher fertilization rates [76]. However, other groups could not replicate these results [77,78], and lately, a PAWP-null mouse model has been shown to induce normal Ca^2+^ release and successful fertilization [79]. At present, there is abundant and valuable evidence showing that PLCζ represents the main SOAF. The first strong evidence came from Saunders et al. 2002, who detected PLCζ as a novel sperm-specific PLC isoform [80]. It was additionally demonstrated that the removal of PLCζ from sperm extracts abolished Ca^2+^ release and that the injection of PLCζ cRNA into mouse oocytes induced Ca^2+^ oscillations similar to those observed after fertilization [80]. In the following years, many studies confirmed these results and reported the induction of Ca^2+^ oscillations after the injection of PLCζ cRNA [81,82] and human recombinant PLCζ protein [83] into human oocytes. Moreover, the abolishment of different PLCζ regulatory domains disrupted Ca^2+^ oscillation activity [84] and the reduction of PLCζ in mice by RNA interference affected the activating capacity of the sperm by inducing altered Ca^2+^ oscillations and lower fertilization rates [85]. Finally, the detection of PLCζ mutations in patients with FF after ICSI confirms its crucial role in the fertilization process [40,45,86].

Interestingly, the recent development of PLCζ knockout (KO) mouse models by two different research groups has raised some additional questions. Both Hachem et al. 2017 and Nozawa et al, 2018 showed that PLCζ-null sperm was unable to induce Ca^2+^ oscillations in mouse oocytes after ICSI [87,88], even after the injection of three sperm cells [88]. Following ICSI with PLCζ-null sperm, a very low number of oocytes reached the two-cell stage, which were probably caused by spontaneous activation by the injection procedure itself, with only one reported embryo reaching the blastocyst stage [87]. When performing in vitro fertilization (IVF) with PLCζ-null sperm, Hachem et al. 2017 did not observe calcium oscillations except in one oocyte showing a single Ca^2+^ transient (1/40) [87]. By contrast, Nozawa et al. 2018 detected that all monospermic fertilized oocytes by IVF showed Ca^2+^ oscillatory activity (28/28) although very abnormal [88]. Following IVF, fertilization rates were comparable between wild-type (WT) and PLCζ KO sperm; however, there was a dramatic increase in polyspermy [87,88], which is likely to be attributed to the insufficient Ca^2+^ release, since PLCζ abolishment did not impair the ability of the sperm to undergo acrosome reaction and to bind to the oocyte [87]. Nozawa et al. 2018 also showed that when two PLCζ-null sperm fused to the oocyte instead of one, the number of Ca^2+^ spikes increased, and more oocytes resumed the cell cycle [88]. Finally, in vivo mating experiments of PLCζ KO mice with WT females showed that PLCζ-null mice are not infertile, but surprisingly subfertile, as they are able to have offspring albeit at lower efficiency [87,88]. Altogether, these results validated the role of PLCζ as the SOAF necessary during ICSI but also pointed to the potential existence of alternative sperm factors or independent oocyte activation mechanisms being active in mouse when PLCζ is absent during IVF and in vivo fertilization. PLCζ-independent oocyte activation mechanism allows the fertilization, embryo development, and live birth with decreased efficiency in comparison to PLCζ-dependent activation, and it seems to be triggered by the interaction between the sperm and oocyte membranes, which is bypassed by the ICSI technique [87,88]. However, little is known about these alternative pathways. It was suggested that the fertilization observed after IVF and in vivo fertilization could have been caused by the spontaneous activation of mouse oocytes, which is a quite common event in some mouse strains that could have been rescued by the presence of the male genome [47,87]. However, it seems more likely that other sperm or oocyte factors rescue the FF caused by PLCζ absence. One possibility is the partial contribution of the previous suggested SOAFs, such as PAWP or tr-kit [73,75]. Alternatively, other sperm and egg PLC isoforms that have been reported in the past to participate in the fertilization process of different species could also be involved [89,90,91,92,93]. For example, the knockdown of PLCβ in mouse oocytes, as well as the injection of PLCβ cRNA, was observed to perturb the Ca^2+^ oscillation pattern, suggesting a combined activity of egg PLCβ with sperm PLCζ for successful oocyte activation [93]. More research is needed to understand whether there is a contribution of other factors when PLCζ is not functional. Studying the role of the previously mentioned proteins in the PLCζ-null mice during IVF would be of huge interest to better understand the complex process of mammalian fertilization.

## 3. Diagnostic Tools to Detect Oocyte Activation Deficiencies Caused by Sperm Factors

When low fertilization or total fertilization failure occurs after ICSI, it is advisable to assess whether it is caused by a sperm- or an oocyte-related factor. Being able to answer this question thoroughly improves patient counseling, allowing doctors to recommend the most appropriate treatment for the next cycle and warn patients about possible genetic transmission to their children when genetic defects are detected. There are a couple of tests available to determine the cause of the oocyte-activating deficiency. These assays directly investigate the male gamete due to its easy accessibility and include the study of the activation rate and Ca^2+^ oscillatory capacity of the spermatozoa (in heterologous and homologous ICSI tests) as well as the evaluation of altered PLCζ presence (by genetic screening, gene expression analysis, and protein localization and quantification assays) [30]. When, according to these tests, the male gamete is proven to be functional, it is assumed that the female gamete is responsible for the fertilization failure. In the following section, a detailed description of the diagnostic tests used to detect sperm factors causing fertilization failure is given.

### 3.1. Heterologous ICSI Tests

The scarcity of human oocytes available for research purposes has made the scientific community develop heterologous ICSI tests using oocytes from different mammalian species (mouse, hamster, rabbit and bovine) to test the fertilizing potential of human spermatozoa [35,94].

Hamster oocytes are known for their high survival rate even with harsh microinjection methods and for their relatively translucent cytoplasm, which permits an easy observation of different events related to fertilization [95]. Therefore, the ICSI of human sperm into hamster oocytes has been used in the past as a model to evaluate sperm head decondensation and male pronucleus formation [96]. The ICSI of human sperm with severe male-factor infertility into hamster oocytes showed a reduced percentage of sperm head decondensation [97]. Moreover, when the hamster-ICSI assay was applied to a patient with normal semen characteristics but who exhibited low fertilization rate after ICSI, the activation rate after injection of a patient’s sperm into hamster oocytes was significantly lower than that of the control sperm (11.6%, *n* = 60% vs. 68.3%, *n* = 60) [98].

Rabbit and bovine oocytes have served as valuable models to study human sperm centrosomal function, since the centrosome is paternally inherited in these species, just as in human [35,99]. Conversely, rodent oocytes cannot be used for this objective, as the centrosome is maternally derived [100,101]. One study has applied the heterologous ICSI of human sperm into rabbit eggs to detect male factor infertility caused by altered centrosomal function, reporting that the average aster formation rate was significantly lower when injecting sperm from infertile patients with low cleavage rates compared to sperm from fertile donors (3.6 ± 2.9%; *n* = 4 vs. 35.0 ± 1.5%; *n* = 2) [102]. Similarly, when performing heterologous ICSI with sperm from infertile patients into bovine eggs, the reported sperm aster formation rate was reduced in comparison to that of the fertile donors (47.0 ± 18.5%; *n* = 15 vs. 66.1 ± 7.2%; *n* = 3) [103]. Overall, the use of bovine oocytes has a preference over the use of rabbit oocytes, as sperm aster formation rates of fertile donors are generally higher [104]. The bovine-ICSI assay has also been used to detect centrosomal abnormalities in patients with globozoospermia [105] and in a case of fibrous sheath dysplasia [106].

Thus far, mouse oocytes are the most commonly used model to study the fertilizing capacity of human sperm. Their major advantages include high cleavage rates obtained after the injection of human donor sperm (>90%), high yields of oocytes obtained per mouse, the relatively low spontaneous activation and the ease in housing and handling due to its small size. A heterologous ICSI of human sperm into mouse oocytes is used to assess the activation rate as well as the calcium oscillation pattern produced after injection of infertile patient sperm in comparison to a fertile donor sperm.

#### 3.1.1. Mouse Oocyte Activation Test (MOAT)

**Figure 2:**
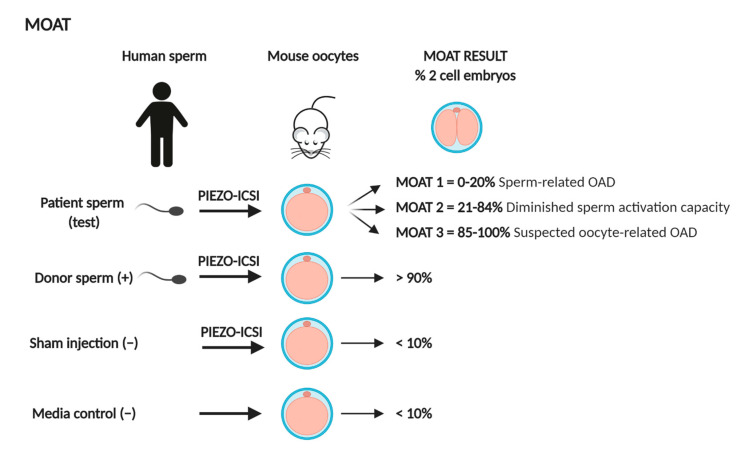
Schematic representation of the mouse oocyte activation test (MOAT). (+): Positive control group (−): Negative control group; PIEZO-ICSI: Piezo-assisted intracytoplasmic sperm injection; OAD: oocyte activation deficiency.

The mouse oocyte activation test (MOAT) consists of the injection of human sperm cells into fresh MII mouse oocytes by piezo-driven ICSI [48,94]. For each MOAT, four groups are established: (i) injection of 40 oocytes with the patient spermatozoa to test; (ii) injection of 40 oocytes with control sperm with proven fertility (positive control); (iii) sham injection of 10 oocytes (negative control); (iv) 10 non-manipulated media control oocytes (negative control). This test enables the classification of the patients in three groups depending on the mouse oocyte activation rate (two-cell formation) in comparison to the fertile control samples [107] (Figure 2). Patients with activation rates lower than 20% are classified as MOAT 1 and diagnosed with a sperm-related oocyte activation deficiency (OAD). Notably, globozoospermic patients are allocated to this group [45,48,107,108]. Globozoospermia has been correlated with reduced levels of PLCζ [44,45,109]. Patients with activation rates between 21% and 84% are classified as MOAT 2. Initially, this group was defined as “inconclusive”, since intermediate activation rates were observed. However, the analysis of the Ca^2+^ patterns obtained after fertilization in this group revealed aberrant or absent Ca^2+^ oscillations, pointing to a sperm-related activation deficiency as well (see Section 3.1.2) [49]. Finally, patients with normal activation rates higher than 85% (similar to the fertile controls), classified as MOAT 3, have a normal sperm-activating capacity; hence, they are diagnosed with a suspected oocyte-related OAD. The test is reliable when the activation rate of the fertile donors is >90% and both the sham injection and medium control show activation rates <10%. Embryos are destroyed immediately after the assessment of the activation rate. In addition, the hybrid embryos do not normally progress beyond the two-cell stage. The MOAT is a valuable diagnostic test to distinguish between male and female factors causing fertilization failure after ICSI and predicts the success of AOA as a treatment option [52,107] (see Section 4.2). Nevertheless, the need of mouse housing facilities, specialized equipment, and personnel with expertise in piezo-driven ICSI, as well as the ethical concerns regarding the use of heterologous ICSI, explains why MOAT has only been used by a few laboratories worldwide [110,111,112,113]. In order to facilitate its application, a recent article has proposed the use of vitrified-warmed mouse oocytes to perform MOAT, instead of fresh oocytes [114]. No significant differences in the mouse oocyte activation rate were found when using vitrified-warmed mouse oocytes in comparison to fresh mouse oocytes (88.5%; *n* = 81 vs. 93.8%; *n* = 78) (*p* > 0.05) [114].

#### 3.1.2. Mouse Oocyte Calcium Analysis (MOCA)

With the aim to facilitate the distinction between sperm-related and oocyte-related deficiencies, which could both contribute to MOAT group 2 (intermediate activation rates), Vanden Meerschaut et al. designed the mouse oocyte calcium analysis (MOCA) at Ghent University Hospital in 2013 [49]. This test consists of the analysis of the Ca^2+^ pattern (hallmark of fertilization) observed after the injection of patient human spermatozoa into MII mouse oocytes. MOCA is based on the quantitative measurement of the free cytosolic Ca^2+^ present in the oocytes by the use of fluorescent probes. Before the piezo-driven ICSI is performed, mouse oocytes are exposed to a Ca^2+^-sensitive fluorescent dye (e.g., Fura-2 AM). Since the plasma membrane of human sperm is physically and biochemically more stable than that of the mouse and disintegrates more slowly when injected into mouse oocytes causing the first calcium oscillation to appear later [115], the human sperm is treated with a plasma membrane disrupting agent (e.g., L-α-lysophosphatidylcholine) prior to the procedure, making the Ca^2+^ imaging procedure less time-consuming.

**Figure 3:**
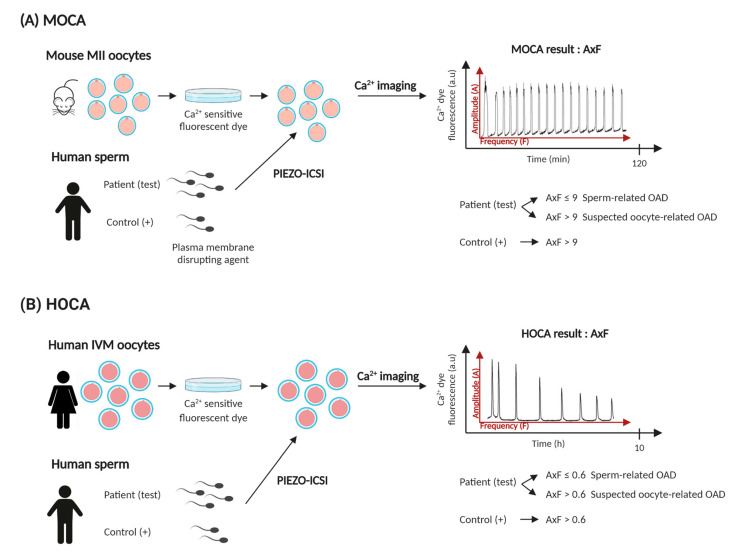
Schematic representation of the diagnostic tests designed to study the calcium (Ca^2+^) oscillation pattern obtained after injection of human sperm into mouse and human oocytes. (**A**) Mouse oocyte calcium analysis (MOCA). (**B**) Human oocyte calcium analysis (HOCA). (+): Positive control group; PIEZO-ICSI: Piezo-assisted intracytoplasmic sperm injection; OAD: oocyte activation deficiency; A: amplitude; F: frequency.

After piezo-driven ICSI, oocytes are placed under an inverted epifluorescence microscope equipped to stabilize standard culture conditions and provide the corresponding excitation wavelengths for the fluorescent probe used. Images are acquired every 5 s during a period of 2 h. The representation of the emitted fluorescence (a.u.) over time corresponds to the Ca^2+^ pattern expressed by each oocyte (Figure 3A). All individual oocytes analyzed are scored depending on the frequency of the Ca^2+^ rises: no Ca^2+^ spike (score “0”), 1–2 spikes (score “+”), 3–10 spikes (score “++”), and more than 10 spikes (score“+++”). The product of the mean frequency (F) and the mean amplitude (A) of all Ca^2+^ patterns of one patient is also calculated and compared to the AxF control value. This product represents the total amount of Ca^2+^ released. When correlating the MOAT groups with the frequency of Ca^2+^ rises observed after MOCA, patients classified as MOAT 1 showed no or very abnormal Ca^2+^ oscillation patterns (with 87.6% of the oocytes scoring “0” or “+”), patients classified as MOAT 2 showed a high degree of variability in the calcium spiking pattern, but it was very different from that of the control patients (50% of the oocytes scored “0” and only 36.7% showed higher frequency scores “++” and “+++”). Finally, the sperm from MOAT 3 patients was able to induce calcium oscillations in the majority of the oocytes (with 72.7% of the oocytes scoring “++” or “+++”). Interestingly, when correlating the MOAT results with the calcium pattern represented as AxF product, a hyperbolic function is obtained. Therefore, low MOAT results also show a low AxF product, while higher MOAT results show a high AxF product. A threshold value has been established to distinguish between sperm- and oocyte-related activation deficiencies. An AxF ≤ 9 indicates a diminished Ca^2+^ inducing capacity and therefore a sperm-related activation deficiency, while a AxF > 9 value points toward normal Ca^2+^ oscillations and therefore, the couple is more likely to suffer from an oocyte-related activation deficiency. Almost all MOAT 2 patients analyzed with MOCA showed an AxF product ≤ 9, which indicated that patients classified in the MOAT 2 group should also be considered as having a sperm-related deficiency [49]. To avoid the dependency on live mouse facilities and reduce the time needed to perform MOCA, the use of vitrified-mouse oocytes instead of fresh oocytes has also been proposed [114]. However, oocyte vitrification compromises the Ca^2+^ signaling machinery with lower amounts of Ca^2+^ being released. Hence, if vitrified-warmed oocytes are used to perform MOCA, the classification criteria need to be adapted to correctly interpret the results [114].

### 3.2. Homologous ICSI Tests

It has been shown that human PLCζ is more effective at producing Ca^2+^ oscillations in mouse oocytes even when expressed at much lower levels than mouse PLCζ [116,117]. This implies that a reduced amount of human PLCζ protein may activate mouse oocytes but may not be enough to activate human oocytes. Thus, the MOAT and MOCA tests, which rely on the use of mouse oocytes, may not be able to reveal all sperm-activating deficiencies. In fact, the first direct evidence that mouse oocytes may not always be informative to detect sperm-borne oocyte activation deficiencies was obtained by Nikiforaki et al. 2014 [118]. One patient diagnosed with normal sperm activation capacity (MOAT 3, 93% mouse oocyte activation rate) was not able to induce Ca^2+^ oscillations after the injection into in vitro matured human oocytes (IVM), pointing toward a sperm-related activating deficiency after all [118].

Ideally, the best diagnostic test to determine the activating capacity of human sperm would be to inject in vivo matured control oocytes (MII oocytes) with the patient sperm and evaluate the fertilization rate and/or the Ca^2+^ oscillation pattern. However, MII oocytes are usually used exclusively for fertility treatment and therefore, they are not accessible for research purposes. Alternatively, IVM oocytes (germinal vesicle (GV) or metaphase I (MI) oocytes) or in vivo matured metaphase II oocytes with smooth endoplasmic reticulum aggregates (SERa oocytes) can be used. Although some articles have reported the use of ICSI into IVM oocytes and the subsequent analysis of fertilization potential to exclude male factor infertility in couples with low fertilization after ICSI [33,119], most countries ban the creation of human embryos for research purposes [120]. A recently published and better approach is the human oocyte calcium analysis (HOCA) [50]. This test determines the Ca^2+^ oscillation pattern obtained after the injection of human sperm into human IVM oocytes. Oocytes are destroyed right after the measurement (10 h post-ICSI) before fertilization is completed (16–18 h post-ICSI).

#### Human Oocyte Calcium Analysis (HOCA)

The HOCA represents the most sensitive diagnostic test for assessing human sperm-activating capacity [50]. This test is based on the MOCA and consists of the exposure of vitrified-thawed donated human IVM or SERa oocytes to a Ca^2+^-sensitive fluorescent dye, prior to the injection with patient human sperm (Figure 3B). After ICSI, the oocytes are subjected to Ca^2+^ imaging (similarly to MOCA). For HOCA, the Ca^2+^ measurements are acquired every 30s for 10 h to ensure the recording of all Ca^2+^ peaks produced. For this reason, a Ca^2+^-sensitive dye that can be retained for a longer period of time without significant loss of fluorescence is required (e.g., FuraPE3-AM). The Ca^2+^ pattern data analysis is performed based on the protocol established by Vanden Meerschaut et al., 2013. First, the observed Ca^2+^ spike frequency per oocyte analyzed is classified in one of the following four categories: (0) total absence of a Ca^2+^ spike, (+) 1–2 spikes, (++) 3–9 spikes, and (+++) ≥ 10 spikes. Secondly, the activation potential per sperm sample is scored as the product of the mean amplitude per mean frequency (AxF) of the total oocytes injected. Ferrer-Buitrago et al., 2018, evaluated the sperm-induced Ca^2+^ oscillatory profile from MOAT 2 (diminished sperm activation capacity) and MOAT 3 (normal sperm activation capacity) patients after the injection into mouse (MOCA) and human oocytes (HOCA). Results showed that both MOCA and HOCA tests of MOAT 2 patients revealed abnormal Ca^2+^ oscillatory patterns, with HOCA showing a more pronounced decrease in the Ca^2+^ spiking activity. Moreover, HOCA revealed that some MOAT 2 patients are totally incapable of producing Ca^2+^ oscillations in human oocytes. In contrast, the majority of MOAT 3 patients showed normal Ca^2+^ oscillatory patterns after MOCA and HOCA, but HOCA demonstrated the incapability of inducing normal Ca^2+^ oscillations in two patients with normal MOCA values [50]. Furthermore, HOCA results were also correlated with the response to ICSI-AOA treatment. Plotting HOCA AxF values against fertilization rates after ICSI-AOA demonstrated that patients with AxF ≤ 0.6 showed abnormal sperm activating capacity and favorable response to ICSI-AOA, while patients with AxF > 0.6 showed normal sperm activating capacity and unfavorable response to ICSI-AOA, pointing to a sole oocyte-related activation deficiency (Figure 3B).

It is noteworthy that procedures such as in vitro maturation, in vitro aging, and cryopreservation affect the Ca^2+^ oscillatory patterns by altering the frequency, amplitude, and duration of the Ca^2+^ spikes, in comparison to the patterns observed after the use of fresh in vivo MII oocytes [66]. In addition, the presence of SERa aggregates in MII oocytes has also been reported to slightly affect the Ca^2+^ oscillatory response [121]. Moreover, culture conditions have also been reported to alter the Ca^2+^ release in mouse and human oocytes [70]. Nevertheless, HOCA performed on vitrified-thawed IVM and SERa oocytes shows higher sensitivity than the MOCA test using fresh mouse oocytes, but its applicability remains challenging in clinical practice, as human oocytes remain scarce for research purposes [50]. Therefore, it is important to search for alternative, more sensitive diagnostic tests. Recently, equine oocytes have been shown to display very similar Ca^2+^ patterns as human oocytes after ICSI [122], and it would be advantageous to test whether equine oocytes may serve as a more sensitive test to detect sperm-related activation deficiencies in patients experiencing FF compared to mouse.

### 3.3. Particle Image Velocimetry (PIV)

One of the drawbacks of performing calcium imaging is that the use of fluorescent dyes and high-intensity excitation light causes damage to the oocytes. Thus, fertilization and embryo development cannot be assessed following Ca^2+^ pattern analysis, which implies that this technique can only be performed in a research or diagnostic setting. Some years ago, a non-invasive time-lapse method to quantify the Ca^2+^ oscillations based on particle image velocimetry (PIV) was proposed [123]. Ajduk et al., 2011, showed that fertilization in mouse oocytes induces rhythmical cytoplasmic movements that correlate with the contractions of the actomyosin cytoskeleton triggered by the Ca^2+^ oscillations. Moreover, it was also reported that the pattern of cytoplasmic movements upon fertilization was associated with the developmental potential of the zygotes, suggesting that this method could be used during clinical settings to evaluate the capability of in vitro fertilized human oocytes to develop into blastocysts [123]. Likewise, rhythmical cytoplasmic movements using PIV were also detected and correlated to the Ca^2+^ oscillations produced after injection of PLCζ cRNA into unfertilized human oocytes [124]. Recently, the analysis of cytoplasmic movements by PIV in combination with morphokinetic analysis of fertilized mouse embryos until day 5 was shown to be an accurate time lapse imaging technique to predict the zygote ability to form a high-quality blastocyst [125]. However, before the application of this technique in IVF laboratories, further validation and risk assessment in human oocytes should be performed.

### 3.4. Techniques to Assess Compromised PLCζ Presence

During the past decade, accumulated evidence has demonstrated that the presence of abnormal PLCζ protein, as well as altered PLCζ levels and localization patterns in sperm cells, causes fertilization failure after ICSI [126,127]. Therefore, the assessment of PLCζ in human sperm has emerged as a useful and accessible diagnostic tool for all laboratories worldwide, which do not require animal facilities, specialized equipment, or the use of human oocytes.

#### 3.4.1. Genetic Screening

**Figure 4:**
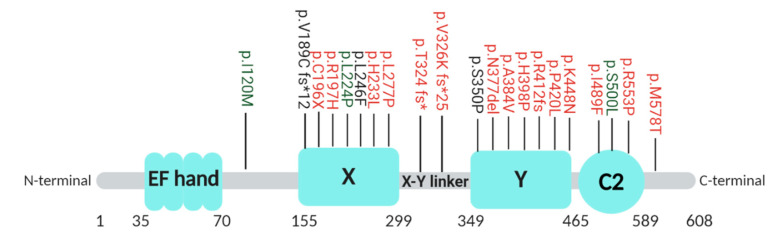
Overview of the localization of the identified mutations in PLCζ. The amino acid sequence position of the protein domains and the identified mutations are indicated by the numbers. In red, mutations linked to male infertility by functional analysis. In green, mutations not associated to male infertility by functional analysis. In black, mutations not studied by functional analysis yet.

The *PLCZ1* gene (ENSG00000139151) is located on chromosome 12 and consists of 15 exons, which forms a protein with four different regulatory domains: four tandem EF hand domains at the N-terminus (necessary for Ca^2+^ sensitivity), a catalytic X and Y domain in the middle of the protein (important for PIP_2_ hydrolysis and substrate binding), and a C2 domain at the C-terminus (involved in substrate binding to PI_3_P and PI_5_P membrane phospholipids) [38]. PLCζ specifically lacks the Src homology (SH) domain and Pleckstrin homology (PH) domain, which makes it the smallest PLC isozyme known with a protein length of 608 amino acids and molecular mass of 70 kDa [128]. Up to now, 21 different mutations in the *PLCZ1* gene have been reported to cause low or total fertilization failure after ICSI [40,41,45,71,86,129,130,131,132,133,134] (Table A1). These mutations have been found throughout all regulatory regions of the PLCζ protein (Figure 4), affecting the protein function in different manners.

All mutations have been assessed by in silico analysis, and the majority did undergo functional analysis as well (Figure 4, Table A1). Functional analysis can be performed by injecting mutant human PLCζ cRNA (or the corresponding mouse mutant PLCζ cRNA) either into mouse MII or human IVM oocytes. An interpretation of cRNA injection experiments might be complicated by the used concentration of PLCζ cRNA. For example, Torra et al., 2019, performed the functional analysis with a concentration of 100 ng/µl PLCζ cRNA [40], while Yan et al., 2020, used a concentration of 300 ng/µL [41]. As a future reference, injection of 100 ng/µL PLCζ cRNA has been suggested as the optimal concentration to obtain comparable Ca^2+^ oscillation patterns and activation rates to the ones obtained after injection of human sperm into IVM human oocytes [82].

Only one mutation present in heterozygosis has been found in the EF-X linker region (p.I120M) in a patient suffering from total fertilization failure (0/18 fertilized oocytes) [40]. However, both in silico and functional analysis by injecting human PLCζ cRNA into human IVM oocytes showed no effect on PLCζ competence to trigger oocyte activation, suggesting that perhaps a second, non-identified mutation in deep-intronic or regulatory regions of *PLCZ1* contribute to the observed phenotype or that other sperm or oocyte factors are involved [40].

Seven different mutations have been found in the X catalytic domain (p.V189C fs*12; p.C196*; R197H; p.L224P; p.H233L; p.L246F; p.L277P), as well as in the Y catalytic domain (p.S350P; p.N377del; p.A384V; p.H398P; p.R412fs; p.P420L; p.K448N); some of them have been identified in different studies. Most of these mutations are predicted by in silico analysis to be detrimental, as they interfere with the secondary structure of the catalytic center of the protein and thus are expected to affect the enzymatic activity. Functional analysis by PLCζ cRNA injection reveals variability in the effect of each mutation in the fertilization rates when comparing the result to the wild-type PLCζ cRNA (Table A1).

The X-Y linker region is associated with the presence of positively charged amino acids, which has been reported to be essential for binding the substrate PIP_2_ [129]. Two frameshift mutations have been found in the X-Y linker region (p.T324 fs and p.V326K fs*25), which alters the reading frame and thus amino acids present in this region, as well as generates stop codons resulting in the loss of the Y and C2 domain of PLCζ, profoundly affecting the protein activity [40,130].

Three proteins predicted to be damaging have been found in the C2 domain (p.I489F, p.S500L, p.R553P). While p.I489F and p.R553P result in reduced oocyte activation rates after an injection of mutant human PLCζ cRNA into mouse and IVM human oocytes, respectively [86,131], p.S500L does not influence fertilization rates when injected into IVM human oocytes [40]. However, the latter mutation has been detected in 9/37 (24.3%) patients with fertilization failure analyzed by the same study [40], compared to its presence in 3% of the general population (minor allele frequency reported by the ExAc database) which suggests its direct association to the phenotype. Perhaps p.S500L does not result in lower activation rates because the amount of human PLCζ cRNA injected (100 ng/µL) exceeds physiological levels, because the Ca^2+^ patterns induced are altered due to the use of IVM oocytes or because other sperm factors are also involved.

Finally, one mutation has been found outside the protein domains in the C-terminal region (p.M578T). Mutant PLCζ cRNA injection into human IVM oocytes did not result in pronuclear formation [41].

Most of the patients present with homozygous or compound heterozygous *PLCZ1* mutations (Table A1). However, Torra et al., 2019, detected almost all mutations in heterozygosis, even in some patients with total FF. It is hypothesized that this effect is due to haploinsufficiency [40]. As the sperm cells are haploid, one would consider that a patient with a heterozygous *PLCZ1* mutation would have only half of his sperm cells affected and consequently, approximately half of the injected oocytes would fail to fertilize after ICSI. However, the translation of mRNA halts at the primary spermatocyte stage (diploid) [132]. Thus, an individual harboring a heterozygous *PLCZ1* mutation would carry 50% mutant and 50% WT protein in his spermatocytes. Then, these proteins will be randomly distributed to the secondary spermatocytes (haploid stage) after meiosis I, giving rise to sperm cells with a reduced but variable amount of functional PLCζ protein, which is insufficient to obtain proper fertilization, and this is independent of the presence of a mutant or WT *PLCZ1* allele at the DNA level. This could partly explain why some heterozygous individuals can conceive naturally, such as men that had fathered homozygous individuals [41,133]. In these cases, by chance, certain sperm cells with a reduced but sufficient amount of WT protein can contribute to successful fertilization. However, the possibility that other sperm factors come into play in reaction to the reduced absence of functional PLCζ cannot be excluded. Moreover, the capacitation process and acrosome reaction bypassed by ICSI may be necessary to support these secondary sperm factors or activate the oocyte by other mechanisms than Ca^2+^ oscillations. In line with these intriguing questions, it still remains to be solved why the sperm from the previously mentioned PLCζ KO mice cannot produce Ca^2+^ oscillations after ICSI, but the PLCζ KO mice are subfertile [87,88]. Finally, the fact that the genetic screening of *PLCZ1* only results in the identification of mutations in a subset of patients showing low or total fertilization failure also points to the potential involvement of other factors. Yet, some studies have already performed genetic screening by whole exome sequencing (WES) on this patient population and failed to identify mutations in exonic regions of other potentially relevant proteins. Future genetic screening approaches could also include regulatory sequences upstream of *PLCZ1* sequence and all intronic regions, or alternatively, whole genome sequencing could be performed.

Lastly, the possibility remains that fertilization failure in these patients is caused by oocyte factors. Recently, mutations in the female genes *WEE2*, *PATL2*, *TUBB8,* and *TLE6* have also been linked to fertilization failure [33,134,135,136]. Therefore, it is interesting to establish targeted gene panel screening in couples with fertilization failure. If mutations are discovered, genetic counseling can be provided to the patients regarding the risk of transmission of infertility to their offspring.

#### 3.4.2. Gene Expression Analysis, Protein Localization, and Quantification Assays

Other possible diagnostic tests to determine PLCζ presence are gene expression analysis by quantitative real-time PCR (qPCR), and protein quantification and localization assays by immunostaining and immunoblotting. Aghajanpour et al., 2011, measured the expression of PLCζ mRNA by qPCR in individuals with previous low or total FF after ICSI and individuals diagnosed with globozoospermia. The data revealed that the expression of PLCζ was significantly lower in these groups of patients in comparison to individuals with high fertilization rates after IVF and ICSI (>70%) [137]. In agreement with these results, Tavalaee et al., 2016, also reported lower levels of PLCζ mRNA expression in patients with globozoospermia [138]. Although a reduction of PLCζ mRNA expression may reflect low levels of PLCζ protein, which would explain the male infertility, the exact role of PLCζ mRNA and how it represents the PLCζ protein level in the sperm is not known yet. For this reason, protein quantification assays are a better approach and have been used more frequently. Immunoblotting of PLCζ protein in sperm from patients with low or total FF after ICSI showed an absence or reduced levels of PLCζ in comparison to fertile controls, while immunostaining revealed that the low fertilization rates were also linked to altered localization patterns of PLCζ [41,45,71,86,139,140,141,142]. In fertile individuals, PLCζ normally localizes to the equatorial region of the sperm or is distributed between the equatorial and acrosomal/postacrosomal regions [143], but in infertile patients, this distribution is altered, and PLCζ is usually absent in the equatorial region [45,86,133,139]. Moreover, in globozoospermic patients, PLCζ has been repetitively proved to be lacking, which explains the lower fertilization rates obtained in these individuals [44,45,144]. Nevertheless, there is still controversy on the correlation between PLCζ protein levels and localization pattern and the fertilization capacity of the sperm, as some authors did not find an association [40,145]. Moreover, other articles showed a large degree of variability between PLCζ expression and localization in both the control and patient populations, although the total levels of PLCζ were found to be higher in the control than in the patient group [146]. In another article from the same group, it was observed that the total PLCζ levels, localization patterns, and the proportion of sperm exhibiting PLCζ were significantly correlated with fertilization rates following ICSI, but not IVF [142]. This contradiction and variability in the results may be partially explained by the specificity of the polyclonal antibodies used. A more recent article reported that the use of different antigen unmasking (AUM)/retrieval protocols enabled an enhancement of PLCζ epitope availability and therefore a visualization of PLCζ fluorescence [147]. When analyzing PLCζ expression in sperm from fertile donors with and without AUM, AUM significantly changed the relative PLCζ fluorescence observed [147]. Taking into account these results, the authors suggest that previous studies analyzing PLCζ presence should re-evaluate the results using AUM protocols. Furthermore, the development of a monoclonal antibody for PLCζ would be of great help to obtain more consistent results. Up to now, the predictive potential of PLCζ immunostaining and immunoblotting was questionable. However, two very recent articles have proposed new algorithms using PLCζ immunostaining assay for the clinical management of patients suffering from FF after ICSI, which appear to be very useful to determine the responsible gamete and to predict the efficacy of AOA in the next treatment [51,148].

## 4. Treatment Options for Patients Suffering from Fertilization Failure

Over the past decades, assisted oocyte activation (AOA), the artificial induction of Ca^2+^ oscillations in the oocyte has emerged as an efficient ART to treat couples suffering from low or total FF after ICSI [9,24]. However, the lack of a standardized AOA protocol makes the comparison of results among clinics difficult. Moreover, few studies have been reported on the safety of the technique [53]. For these reasons, ICSI in combination with AOA is still considered as an experimental treatment. The following section summarizes the state-of-the-art knowledge regarding the AOA treatment.

### 4.1. Assisted Oocyte Activation Methods: Efficacy and Safety

The artificial activation of human oocytes can be achieved by three main strategies: electrical, mechanical, or chemical [24,149]. The electrical AOA method involves the application of a high-voltage electrical field to the oocyte, which promotes the formation of pores in the plasma membrane and facilitates the entrance of calcium from the extracellular milieu. Electrical oocyte activation has been successfully applied to treat fertilization failure after ICSI [150,151,152] and also globozoospermia [112], as live births have been reported. The mechanical AOA method is a modified ICSI technique that consists of vigorous aspiration of the cytoplasm while performing ICSI. The repeated dislocation of the ooplasm allows the entrance of the calcium from the extracellular medium and the release of calcium from the broken endoplasmic reticulum. Normal fertilization and embryo developmental rates have been obtained after the use of this technique in comparison to previous ICSI cycles to treat both sperm and oocyte activation deficiencies, and live births have been reported [110,153].

Thus, the chemical AOA method is far most frequently used, since it gives the highest activation and blastocyst formation rates compared to the other AOA strategies. Different activating agents are available, which can be grouped according to the calcium response induced after oocyte activation: a single Ca^2+^ transient, multiple Ca^2+^ oscillations, or the absence of Ca^2+^ oscillations [149].

The Ca^2+^ ionophores, Ionomycin and Calcimycin (GM508 or A23187), are the most common chemical agents used in the IVF clinics for AOA. These soluble lipids confer permeability to the oocyte plasma membrane and allow the transport of Ca^2+^ ions to the cytoplasm. The Ca^2+^ ionophores elicit single Ca^2+^ transients in the oocytes, and although they do not mimic the physiological calcium release during normal fertilization (where multiple calcium oscillations are observed), they are very efficient in triggering oocyte activation. AOA using Ca^2+^ ionophores has been reported to restore fertilization rates and achieve pregnancy in patients with FF after ICSI [48,52,107,154,155] and more specifically to treat male infertility caused by *PLCZ1* mutations [40,41,130,133]. Ca^2+^ ionophore treatment was also beneficial to treat patients with globozoospermia [107,108,156] and severe teratozoospermia [157,158]. The efficacy of Ionomycin versus Calcimycin (A23187) has been studied. Interestingly, Ionomycin was shown to induce higher Ca^2+^ release and consequently higher fertilization rates than Calcimycin in both mouse and human oocytes [159]. These results agree with previous reports suggesting that oocyte activation is triggered when the total dose of Ca^2+^ released reaches a minimal threshold [67]. Thus, the chemical agent selected for AOA, as well as the protocol used (concentration, time, and duration of exposure) influence the total amount of Ca^2+^ discharged, which may explain the variation in efficacy reported by different studies. Determining the minimal required amount of Ca^2+^ release to induce oocyte activation would be of great interest to optimize and design a standardized AOA protocol.

In addition to ionophores, the use of Strontium Chloride (SrCl_2_) as an activating agent has been of great interest. SrCl_2_ is widely accepted to be the most efficient method to induce oocyte activation in mouse oocytes, since multiple Ca^2+^ transients are produced and high blastocyst formation rates are achieved [160,161]. In mouse, the transient receptor potential cation channel, subfamily V, vanilloid 3 (TRPV3), has been identified to mediate Sr^2−^ induced oocyte activation [162]. The agonists of TRPV3 channels (2-aminoethoxydiphenyl borate (2-APB) and carvacrol) have been proven to activate TRPV3 and consequently induce mouse oocyte activation. In human, the efficacy of SrCl_2_ to induce oocyte activation is still under debate. Lu et al., 2018, showed that regarding the presence of functional TRPV3 channels in human oocytes and the efficacy of 2-APB and carvacrol to induce a single Ca^2+^ transient, the exposure of SrCl_2_ did not induce any single calcium rise nor oocyte activation [163]. Nonetheless, some authors have described SrCl_2_ as an efficient AOA method to overcome FF, and live births have been accomplished [164,165,166,167]. This divergence reveals the need to better understand the mechanism of action of SrCl_2_.

Finally, fertilization has also been induced in the absence of Ca^2+^ oscillations, highlighting the idea that Ca^2+^ release may not be indispensable for oocyte activation. Indeed, the release of the oocyte from the meiotic arrest is achieved when the MPF complex is degraded (Figure 1) [4]. Thus, targeting different components that directly influence the MPF levels in the cell are also valid strategies to provoke oocyte activation. For example, downstream CAMKII, a Zn^2+^ spark is required for MPF degradation [168]. It has been reported that reducing the Zn^2+^ level in the oocyte by Zn^2+^ chelators (e.g., N,N,N′ ,N′ -tetrakis(2-pyridylmethyl)ethane-1,2-diamine (TPEN)) can induce oocyte activation in the absence of Ca^2+^ discharge [169]. Other examples are puromycin [170], cycloheximide [171], and roscovitine [64,172]. The first two protein inhibitors block cyclin B synthesis, while the latter inhibits CDK1 (Figure 1), thus inducing MPF degradation and subsequently oocyte activation.

In the past years, research on AOA methodologies has focused on the development of a more physiological and endogenous oocyte activating agent. It has been repetitively shown that an injection of human PLCζ cRNA is able to induce oocyte activation in mouse MII [116] and human IVM oocytes [82]. However, cRNA injection is not an appropriate strategy for clinical treatment due to the risk of uncontrolled expression and the possibility of reverse transcription to cDNA that could be subsequently integrated in the oocyte’s genome [24]. Therefore, a purified and stable recombinant human PLCζ (rhPLCζ) protein would be the best alternative to rescue oocyte activation, especially in patients where the FF can be attributed to a PLCζ deficiency. Different rhPLCζ have been produced [83,140,173] and shown to be efficient in triggering Ca^2+^ oscillations when injected into mouse and human oocytes. In addition, it has been reported that failed egg activation after an injection of mutant PLCζ cRNA could be rescued by the subsequent injection of rhPLCζ, leading to successful embryo development to the blastocyst stage [173].

**Figure 5:**
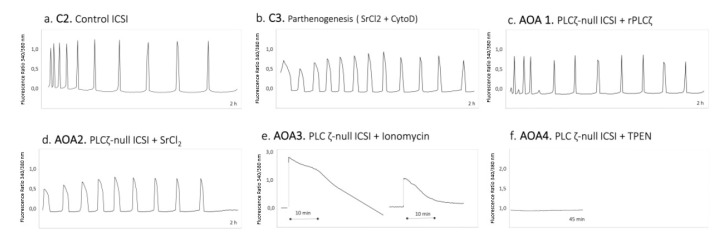
Representative Ca^2+^ signaling responses registered after in vitro fertilization in mouse oocytes in combination with different assisted oocyte activation methodologies. (**a**) Physiological Ca^2+^ oscillation pattern obtained after an injection of wild-type sperm into mouse oocytes (Control 2). (**b**) Ca^2+^ oscillation pattern observed after the parthenogenetic activation of mouse oocytes using SrCl_2_ in combination with Cytochalasin D (Control 3). (**c**–**f**) In order, Ca^2+^ oscillation patterns obtained after injection of PLCζ-null sperm in combination with rhPLCζ (AOA1), SrCl_2_ (AOA2), Ionomycin (AOA3), and the Zn^2+^ chelator TPEN (AOA4). Reproduced from Minerva et al., 2020 with permission [174].

Recently, a comprehensive study was performed in mouse, comparing pre-implantation development following four different relevant AOA methodologies (rhPLCζ, SrCl_2_, Ionomycin, and TPEN), using PLCζ-null mouse sperm [174]. Ferrer-Buitrago et al., 2020, reported that different AOA methods, each of which induces different Ca^2+^ profiles (Figure 5), result in comparable oocyte activation rates but different efficacy regarding embryo development. AOA methods that induced Ca^2+^ oscillatory responses (rhPLCζ and SrCl_2_) or single Ca^2+^ transients (Ionomycin) showed no significant difference on the blastocyst formation rate in comparison to ICSI controls, but the AOA method inducing oocyte activation in the absence of Ca^2+^ oscillations (TPEN) shows reduced compaction and blastocyst formation rates. It is worth mentioning that SrCl_2_ gave the highest blastocyst formation rate (75%), and that rhPLCζ and Ionomycin showed comparable results (41% vs. 50% respectively). Even though these results point toward the favorable use of rhPLCζ, the method of preference will remain the use of Ca^2+^ ionophores until rhPLCζ is commercially available.

The use of artificial agents to induce oocyte activation requires caution [175]. The exposure of oocytes to AOA agents (for a short time) may cause cytotoxic effects or epigenetic alterations. Some animal studies investigating the safety of different AOA strategies on the pre-implantation and post-implantation development have been published and reported reassuring results. Transcriptional analysis did not detect differences in gene expression profiles between blastocysts derived from in vivo fertilization, control ICSI and AOA using rhPLCζ, SrCl2, Ionomycin, and TPEN [174]. Moreover, pups born after AOA treatment with SrCl_2_, Ionomycin, and electrical pulses did not show any abnormalities at birth or during early development and were fertile, as they mated normally obtaining healthy offspring [161]. However, these results should be extrapolated with caution to human, and follow-up studies of children born after AOA are required. For now, a few articles reported encouraging results, but the sample size of these studies remain low. A study performed at the Department for Reproductive Medicine, Ghent University Hospital evaluated the neonatal and neurodevelopmental outcome of 21 children born after AOA using Ionomycin and could not detect any serious adverse effects [176]. Other reports have also not observed differences in the physical or mental health of infants born after ICSI and ICSI in combination with AOA using Ionomycin [177,178,179], Calcimycin (A23187) [178,180], and Srcl_2_ [180,181]. Finally, the effect of AOA on chromosome segregation errors has also been evaluated in human oocytes and no increase on aneuploidy rates after the use of AOA was detected [182]. Although altogether, these data support the safety of the AOA treatment, it is still prelaminar and more research should be conducted. Thus, for now, AOA treatment should be considered as an experimental technique.

### 4.2. AOA Efficacy to Overcome Fertilization Failure Caused by Sperm Versus Oocyte Factors

At present, AOA is mostly routinely used to treat fertilization failure after ICSI. Even though it has been reported as a beneficial treatment, differences are observed when comparing the efficacy of AOA to treat fertilization failure caused by either sperm or oocyte factors. A robust study performed by Bonte et al., 2019, compared the results of AOA treatment in patients with OAD over a period of 17 years at the Department for Reproductive Medicine, Ghent University Hospital [52]. In this center, the MOAT test (see Section 3.1.1) is applied to all patients with previous FF after ICSI, and AOA is performed by the injection of calcium chloride (CaCl_2_) together with the patient sperm at the moment of fertilization, followed by a 2-fold ionomycin exposure (10 µg/µL) 30 min apart. When comparing the effect of AOA for the different MOAT groups, a beneficial effect was seen for all groups. Fertilization rates increased significantly from 9.7%, 14.8%, and 17.7% after conventional ICSI to 70.1%, 63.0%, and 57.3% after AOA, while live birth rates increased from 0.0%, 2.8%, and 0.0% after conventional ICSI to 41.2%, 22.6%, and 22.1% after AOA, for MOAT group 1, 2, and 3 respectively. However, MOAT group 1 (sperm-related oocyte activating deficiency) showed significantly higher fertilization rates than MOAT group 2 (diminished sperm oocyte activating capacity), and the latter showed significantly higher fertilization rates than MOAT 3 (normal fertilization capacity of sperm, suspected oocyte activation capacity). In accordance with these results, other studies also reported that patients with oocyte factors show a less beneficial response to AOA treatment [32,50,54].

The MOAT, MOCA, and HOCA are very helpful tests to predict the potential efficacy of an AOA treatment [49,50,52,107]. When the patient is classified as MOAT group 1, a sperm oocyte activating deficiency is confirmed, and AOA is predicted to be very beneficial. If MOCA and HOCA are performed in this group of patients, Ca^2+^ oscillations patterns are absent or aberrant. Thus, Ca^2+^ imaging is not of added value for MOAT group 1. When the patient is classified as MOAT 2, a MOCA test is useful to further confirm the sperm-activating deficiency. In fewer cases, normal oscillations are seen after MOCA. For these patients, an HOCA test may help in revealing subtle sperm factors not detected by mouse assays. Finally, when the patient is classified as MOAT 3, HOCA represents the most precise test to uncover subtle sperm factors not detected by mouse assays or to confirm the presence of an oocyte factor (Table 1). For MOAT 3 patients where HOCA confirms the oocyte factor, ICSI-AOA outcome is very variable. We hypothesize that when ICSI-AOA works, there may be a deficiency in oocyte factors involved in the Ca^2+^ releasing machinery, but when ICSI-AOA fails, it is likely that proteins acting downstream the Ca^2+^ rises are abnormal, thus preventing the oocyte activation process from being completed correctly. Additionally, cytoplasmic immaturity and nuclear defects may also be the cause of FF after AOA treatment. Interestingly, when assessing the Ca^2+^ releasing machinery in failed-to-fertilize oocytes after ICSI-AOA from a patient with normal sperm activating capacity determined by HOCA (AxF = 23.7), it was shown that the oocytes were able to sustain normal Ca^2+^ oscillation patterns after the injection of control sperm [183] supporting our previous hypothesis. Additionally, AOA could not overcome FF in patients with *WEE2* mutations [119,184]. As explained before, WEE2 is an oocyte kinase involved in the release from MII arrest that acts downstream of the Ca^2+^ oscillations. For patients with a confirmed oocyte factor where AOA fails to restore fertilization, there are no alternative ART treatments available to achieve genetic parenthood, and oocyte donation is the only treatment option left. In this context, AOA using targeting molecules that perform their action after the Ca^2+^ oscillations, such as TPEN [169] or roscovitine [64], may be helpful.

Recently, AOA has also been proposed to treat other infertility conditions. Based on the studies showing that altered Ca^2+^ oscillations (an excess or reduction) might have long-term effects on the preimplantation embryo development [68,69,70], AOA was proposed to treat patients suffering from embryo developmental arrest. Ebner et al., 2015, showed that in this group of patients, AOA treatment increased both the blastocyst formation rates and live birth rates (in comparison to the previous ICSI cycle) [185]. Interestingly, in a patient with previous molar pregnancies, AOA was also beneficial, and two healthy deliveries were achieved [118]. Nikiforaki et al., 2016, demonstrated that the patient partner’s sperm could not trigger the normal pattern of calcium oscillations in both mouse and human oocytes and speculated that this low fertilizing ability might had caused insufficient Ca^2+^ release for the normal block to polyspermy allowing dispermic fertilization. Finally, a new retrospective cohort study tested the efficacy of AOA for a variety of infertility conditions: male factor infertility caused by oligoastenoteratozoospermia (OAT), female factor infertility caused by advanced age, unexplained cause, polycystic ovarian syndrome (PCOS), or primary ovarian insufficiency (POI) and a combination of the above factors [186]. After AOA treatment, fertilization rates were significantly increased only in the OAT group, while blastocyst formation rates were significantly increased in patients with OAT, female unexplained infertility, and couples with combined factors. Altogether, these data suggest that AOA may help in overcoming other infertility conditions that are partially caused by altered calcium release, but more evidence is definitely necessary to confirm these results.

## 5. Conclusions

Although PLCζ deficiency has been confirmed as a clear cause of FF after ICSI, it seems undeniable that other sperm factors or alternative oocyte activation mechanisms are contributing to the oocyte activation process as well, at least during IVF and in vivo fertilization. Different lines of evidence support this statement. First, PLCζ KO mice fail to produce Ca^2+^ oscillations after ICSI, but fertilization and normal embryo development can occur after IVF and in vivo fertilization [87,88]. Secondly, human clinical data show that males with heterozygous mutations in PLCζ can suffer from FF after ICSI [40], but that indeed, some heterozygous individuals are fertile during natural fertilization [41,130,133]. Thirdly, not all patients with normal sperm parameters and low fertilization rates after ICSI present with altered PLCζ expression or damaging mutations [40,41,133], even when heterologous ICSI tests point to a sperm-related oocyte activation deficiency. Future research should address these issues that for now cannot be fully explained.

Fertilization failure after ICSI can also be caused by female factors [4]. Therefore, the use of diagnostic tests to distinguish the origin of the infertility is of utmost importance. The heterologous (MOAT and MOCA) and homologous (HOCA) ICSI diagnostic tests show high predictive potential to detect sperm factors and predict the efficacy of subsequent AOA treatment [49,50,107]. However, they are difficult to implement in the IVF labs. In this regard, a combination of genetic screening and immunostaining of PLCζ represents a more simplified and accessible test that will help detecting sperm factors causing male infertility-related FF more rapidly [40,51]. The development of tests that directly investigate the female gamete would also be advantageous, such as genetic screening of female genes related to FF (e.g.,*WEE2, PATL2, TUBB8, TLE6*) [33,134,135,136] or the study of the Ca^2+^ oscillatory machinery in IVM oocytes from the patient after injection of control sperm [183].

Finally, AOA using Ca^2+^ ionophores is a beneficial treatment for male infertility-related FF, and although more studies are required to confirm the safety of the technique, no serious adverse effects have been reported yet [52,176]. However, the development of a commercially available rhPLCζ protein would allow a more physiological AOA strategy and reduce the safety concerns regarding these techniques [173]. For those cases where female factors contribute to the observed FF and AOA fails to restore fertilization, AOA strategies targeting MPF degradation independent of the production of Ca^2+^ oscillations [64,169] may offer possible treatments to achieve genetic parenthood. Finally, AOA treatment should still be considered as an experimental technique and applied with caution only to treat couples with clear oocyte activation deficiencies. More data should be obtained to support preliminary results on the application of AOA to treat couples with other infertility problems.

## Figures and Tables

**Table 1:** Correlation of MOAT, MOCA and HOCA diagnostic test results with ICSI-AOA (assisted oocyte activation) outcomes. The presented data are based on the articles from Heindryckx et al., 2005 [48]; Heindryckx et al., 2008 [107]; Vanden Meerschaut et al., 2013 [49]; Ferrer-Buitrago et al., 2018 [50]; and Bonte et al., 2019 [52].

MOAT Group (OA Rate, %)	MOCA (AxF Value)	HOCA (AxF Value)	Diagnosed OAD (Sperm or Oocyte Factor)	ICSI-AOA Outcome (FR and LBR)
MOAT 1: 0–20%	<9 Absence or very abnormal number of Ca^2+^ oscillations	<0.6 Absence of Ca^2+^ oscillations	Sperm-related OAD	AOA very beneficial (restores FR to normal values ≈70% and increases LBR)
MOAT 2: 21–84%	<9 Abnormal number of Ca^2+^ oscillations	<0.6 Absence or very abnormal number of Ca^2+^ oscillations	Sperm-related OAD	AOA very beneficial (restores FR to normal values ≈70% and increases LBR)
	>9 Normal number of Ca^2+^ oscillations	<0.6 Absence or very abnormal number of Ca^2+^ oscillations	Diminished sperm activating capacity not detected by mouse assays	AOA beneficial (increases significantly FR to ≈60% and improves LBR)
MOAT 3: 85–100%	>9 Normal number of Ca^2+^ oscillations	<0.6 Absence or very abnormal number of Ca^2+^ oscillations	Diminished sperm activating capacity not detected by mouse assays	AOA beneficial (increases significantly FR to ≈50% and improves LBR)
		>0.6 Normal number of Ca^2+^ oscillations	Normal sperm activating capacity, thus oocyte-related OAD	ICSI-AOA outcome very variable. When ICSI-AOA fails to restore FR, patients must be advised for oocyte donation.

OA: oocyte activation, A: amplitude, F: frequency, OAD: oocyte activation deficiency; FR: fertilization rate; LBR: lives birth rate.

## Appendix A

**Table A1:** PLCZ1 mutations detected in patients with low or total fertilization failure after ICSI.OD: outside domain; OA: oocyte activation; IVM: in vitro matured oocytes; C: compound; h: human; m: mouse; cRNA: complementary RNA; NA: not available.

cDNA Change	Localization (Exon)	Protein Change	Localization (Domain)	Mutation Type	Zygosity	PLCζ Level and Localization in Sperm	Predicted Effect by in Silico Analysis	Observed Phenotype by Functional Analysis	References
c.360C > G	4	p.I120M	EF-X linker	Missense	Heterozygous	No difference with control sperm	No effect	No effect on OA after hPLCZ1cRNA injection into human IVM oocytes	[40]
c.570 + 1G > T	5	p.V189C fs*12	X	Splicing	C. Heterozygous	Reduced expression	Damaging. Deletion and premature termination codon	NA	[41]
c.588C > A	6	p.C196*	X	Nonsense	Homozygous/ C. Heterozygous	Reduced expression and altered localization	Damaging Premature termination codon	Absence of expression in HEK293T cells. Reduced OA after hPLCZ1 cRNA injection into mouse oocytes.	[41,130,133,139]
c.590G > A	6	p.R197H	X	Missense	Homozygous/ C. Heterozygous	No difference with control sperm	Damaging	Reduced expression in HEK293T cells. Reduced OA after hPLCZ1 cRNA injection into mouse and human IVM oocytes	[40,130,145]
c.671T > C	6	p.L224P	X	Missense	Heterozygous	No difference with control sperm	Damaging	No effect on OA after hPLCZ1 cRNA injection into human IVM oocytes	[40]
c.698A > T	6	p.H233L	X	Missense	Homozygous/ C. Heterozygous	No difference with control sperm	Damaging	Altered Ca^2+^ patterns and absence of OA after hPLCZ1 cRNA injection into mouse oocytes. Reduced OA after hPLCZ1 cRNA injection into IVM human oocytes.	[40,145,187]
c.736C > T	7	p.L246F	X	Missense	Homozygous	Reduced expression and altered localization	Damaging	NA	[133]
c.830T > C	7	p.L277P	X	Missense	C. Heterozygous	Reduced expression	Damaging	Reduced OA after hPLCZ1 cRNA injection into human IVM oocytes	[41]
c.972_973delAG	9	p.T324 fs	XY-linker	Frameshift deletion	C. Heterozygous	NA	NA	Truncated protein expression in HEK293T cells. Reduced OA after hPLCZ1 cRNA injection into mouse oocytes.	[130]
c.972_973delAG	9	p.V326K fs*25	XY-linker	Frameshift deletion	Heterozygous	No difference with control sperm	Damaging Truncated protein	Reduced FR after hPLCZ1 cRNA injection into human IVM oocytes	[40]
c.1048T > C	10	p.S350P	Y	Missense	Homozygous	Reduced expression and altered localization	Damaging	NA	[133]
c.1129_1131deAAT	10	p.N377del	Y	Frameshift deletion	C. Heterozygous	Reduced expression	Damaging Disruption of the catalytic domain	Absence of OA after hPLCZ1 cRNA injection into human IVM oocytes	[41]
c.1151C > T	10	p.A384V	Y	Missense	Homozygous	Reduced expression	Damaging	Absence of OA after hPLCZ1 cRNA injection into human IVM oocytes	[41]
c.1193C > A	11	p.H398P	Y	Missense	C. Heterozygous	Reduced expression and altered localization	Damaging	Altered Ca^2+^ oscillation pattern after hPLCZ1 cRNA injection into mouse oocytes	[45]
c.1234delA	11	p. R412fs	Y	Frameshift deletion	C. Heterozygous	NA	NA	Truncated protein expression in HEK293T cells. Reduced OA after hPLCZ1 cRNA injection into mouse oocytes.	[130]
c.1259C > T	11	p.P420L	Y	Missense	C. Heterozygous	NA	Damaging	Reduced expression in HEK293T cells. Reduced OA after hPLCZ1 cRNA injection into mouse oocytes	[130]
c.1344A > T	12	p.K448N	Y	Missense	C. Heterozygous	Reduced expression	Damaging	Reduced OA after hPLCZ1 cRNA injection into human IVM oocytes	[41]
c.1465A > T	13	p.I489F	C2	Missense	Homozygous	Absence of expression	Damaging	Altered Ca^2+^ pattern and reduced OA after mPLCz1 and hPLCZ1 cRNA injection into mouse oocytes	[86]
c.1499C > T	13	p.S500L	C2	Missense	Homozygous/ C. Heterozygous	No difference with control sperm	Damaging	No effect on FR after hPLCZ1 cRNA injection into human IVM oocytes	[40,71,145]
c.1658 G > C	13	p.R553P	C2	Missense	Homozygous	No difference with control sperm	Damaging	Reduced OA after hPLCZ1 cRNA injection into mouse oocytes, but not significantly different from control	[131]
c.1733T > C	14	p.M578T	OD (after C2)	Missense	C. Heterozygous	Reduced expression	Damaging	Absence of OA after hPLCZ1 cRNA injection into human IVM oocytes	[41]

## References

[1] World Health Organization Infertility Definitions and Terminology. https://www.who.int/reproductivehealth/topics/infertility/definitions/en/.

[2] Jungwirth A., Giwercman A., Tournaye H., Diemer T., Kopa Z., Dohle G., Krausz C. (2012). European association of urology guidelines on male infertility: The 2012 update. Eur. Urol..

[3] Agarwal A., Mulgund A., Hamada A., Chyatte M.R. (2015). A unique view on male infertility around the globe. Reprod. Biol. Endocrinol..

[4] Yeste M., Jones C., Amdani S.N., Patel S., Coward K. (2016). Oocyte activation deficiency: A role for an oocyte contribution?. Hum. Reprod. Update.

[5] Vander Borght M., Wyns C. (2018). Fertility and infertility: Definition and epidemiology. Clin. Biochem..

[6] Leaver R.B. (2016). Male infertility: An overview of causes and treatment options. Br. J. Nurs..

[7] Ferlin A., Arredi B., Foresta C. (2006). Genetic causes of male infertility. Reprod. Toxicol..

[8] Miyamoto T., Minase G., Okabe K., Ueda H., Sengoku K. (2015). Male infertility and its genetic causes. J. Obstet. Gynaecol. Res..

[9] Kashir J., Heindryckx B., Jones C., de Sutter P., Parrington J., Coward K. (2010). Oocyte activation, phospholipase C zeta and human infertility. Hum. Reprod. Update.

[10] O’Brien K.L.O., Varghese A.C., Agarwal A. (2010). The genetic causes of male factor infertility: A review. Fertil. Steril..

[11] Krausz C., Degl’Innocenti S., Nuti F., Morelli A., Felici F., Sansone M., Varriale G., Forti G. (2006). Natural transmission of USP9Y gene mutations: A new perspective on the role of AZFa genes in male fertility. Hum. Mol. Genet..

[12] Reijo R., Lee T.Y., Salo P., Alagappan R., Brown L.G., Rosenberg M., Rozen S., Jaffe T., Straus D., Hovatta O. (1996). Diverse spermatogenic defects in humans caused by Y chromosome deletions encompassing a novel RNA-binding protein gene. Hum. Reprod..

[13] Writzl K., Zorn B., Peterlin B. (2005). Copy number of DAZ genes in infertile men. Fertil. Steril..

[14] Baccetti B., Collodel G., Estenoz M., Manca D., Moretti E., Piomboni P. (2005). Gene deletions in an infertile man with sperm fibrous sheath dysplasia. Hum. Reprod..

[15] Yatsenko A.N., Roy A., Chen R., Ma L., Murthy L.J., Yan W., Lamb D.J., Matzuk M.M. (2006). Non-invasive genetic diagnosis of male infertility using spermatozoal RNA: KLHL 10 mutations in oligozoospermic patients impair homodimerization. Hum. Mol. Genet..

[16] Avenarius M.R., Hildebrand M.S., Zhang Y., Meyer N.C., Smith L.L.H., Kahrizi K., Najmabadi H., Smith R.J.H. (2009). Human Male Infertility Caused by Mutations in the CATSPER1 Channel Protein. Am. J. Hum. Genet..

[17] ElInati E., Fossard C., Okutman O., Ghédir H., Ibala-Romdhane S., Ray P.F., Saad A., Hennebicq S., Viville S. (2016). A new mutation identified in SPATA16 in two globozoospermic patients. J. Assist. Reprod. Genet..

[18] Elinati E., Kuentz P., Redin C., Jaber S., Vanden Meerschaut F., Makarian J., Koscinski I., Nasr-Esfahani M.H., Demirol A., Gurgan T. (2012). Globozoospermia is mainly due to dpy19l2 deletion via non-allelic homologous recombination involving two recombination hotspots. Hum. Mol. Genet..

[19] Yu J., Chen Z., Ni Y., Li Z. (2012). CFTR mutations in men with congenital bilateral absence of the vas deferens (CBAVD): A systemic review and meta-analysis. Hum. Reprod..

[20] Palermo G., Joris H., Devroey P., Van Steirteghem A.C. (1992). Pregnancies after intracytoplasmic injection of single spermatozoon into an oocyte. Lancet.

[21] Neri Q.V., Lee B., Rosenwaks Z., Machaca K., Palermo G.D. (2014). Understanding fertilization through intracytoplasmic sperm injection (ICSI). Cell Calcium.

[22] Palermo G.D., O’Neill C.L., Chow S., Cheung S., Parrella A., Pereira N., Rosenwaks Z. (2017). Intracytoplasmic sperm injection: State of the art in humans. Reproduction.

[23] Palermo G.D., Cohen J., Alikani M., Adler A., Rosenwaks Z. (1995). Intracytoplasmic sperm injection: A novel treatment for all forms of male factor infertility. Fertil. Steril..

[24] Vanden Meerschaut F., Nikiforaki D., Heindryckx B., De Sutter P. (2014). Assisted oocyte activation following ICSI fertilization failure. Reprod. Biomed. Online.

[25] Mahutte N.G., Arici A. (2003). Failed fertilization: Is it predictable?. Curr. Opin. Obstet. Gynecol..

[26] Bhattacharya S., Maheshwari A., Mollison J. (2013). Factors associated with failed treatment: An analysis of 121,744 women embarking on their first IVF Cycles. PLoS ONE.

[27] Bhattacharya S., Hamilton M.P.R., Shaaban M., Khalaf Y., Seddler M., Ghobara T., Braude P., Kennedy R., Rutherford A., Hartshorne G. (2001). Conventional in-vitro fertilisation versus intracytoplasmic sperm injection for the treatment of non-male-factor infertility: A randomised controlled trial. Lancet.

[28] Flaherty S.P., Payne D., Matthews C.D. (1998). Fertilization failures and abnormal fertilization after intracytoplasmic sperm injection. Hum. Reprod..

[29] Esfandiari N., Javed M.H., Gotlieb L., Casper R. (2005). Complete Failed Fertiltzation After Intracytoplasmic Sperm lniection-Analysis of 10 Years’ Data. Int. J. Fertil. Womens Med..

[30] Bonte D., Reddy Guggilla R., Stamatiadis P., De Sutter P., Heindryckx B., Horcajadas J.A., Gosálvez J. (2018). Unraveling the Causes of Failed Fertilization after Intracytoplasmic Sperm Injection Due to Oocyte Activation Deficiency. Reproductomics.

[31] Yanagida K., Fujikura Y., Katayose H. (2008). The present status of artificial oocyte activation in assisted reproductive technology. Reprod. Med. Biol..

[32] Combelles C.M.H., Zhu L., Fox J.H., Racowsky C. (2010). Diagnosing cellular defects in an unexplained case of total fertilization failure. Hum. Reprod..

[33] Sang Q., Li B., Kuang Y., Wang X., Zhang Z., Chen B., Wu L., Lyu Q., Fu Y., Yan Z. (2018). Homozygous Mutations in WEE2 Cause Fertilization Failure and Female Infertility. Am. J. Hum. Genet..

[34] Esterhuizen A.D., Franken D.R., Becker P.J., Lourens J.G.H., Müller I.I., Van Rooyen L.H. (2002). Defective sperm decondensation: A cause for fertilization failure. Andrologia.

[35] Terada Y., Nakamura S.I., Morita J., Tachibana M., Morito Y., Ito K., Murakami T., Yaegashi N., Okamura K. (2004). Use of mammalian eggs for assessment of human sperm function: Molecular and cellular analyses of fertilization by intracytoplasmic sperm injection. Am. J. Reprod. Immunol..

[36] Verza S., Esteves S.C. (2008). Sperm defect severity rather than sperm source is associated with lower fertilization rates after intracytoplasmic sperm injection. Int. Braz. J. Urol..

[37] Chaichian S., Tamannaie Z., Rohani H., Ahmadi M., Nasr M.H., Pazouki A., Mehdizadehkashi A. (2015). Relationship between sperm parameters and intracytoplasmic sperm injection outcome. Middle East. Fertil. Soc. J..

[38] Nomikos M., Kashir J., Lai F.A. (2017). The role and mechanism of action of sperm PLC-zeta in mammalian fertilisation. Biochem. J..

[39] Durban M., Barragán M., Colodron M., Ferrer-Buitrago M., De Sutter P., Heindryckx B., Vernaeve V., Vassena R. (2015). PLCζ disruption with complete fertilization failure in normozoospermia. J. Assist. Reprod. Genet..

[40] Torra-Massana M., Cornet-Bartolomé D., Barragán M., Durban M., Ferrer-Vaquer A., Zambelli F., Rodriguez A., Oliva R., Vassena R. (2019). Novel phospholipase C zeta 1 mutations associated with fertilization failures after ICSI. Hum. Reprod..

[41] Yan Z., Fan Y., Wang F., Yan Z., Li M., Ouyang J., Wu L., Yin M., Zhao J., Kuang Y. (2020). Novel mutations in PLCZ1 cause male infertility due to fertilization failure or poor fertilization. Hum. Reprod..

[42] Azad N., Nazarian H., Novin M.G., Farahani R.M., Piryaei A., Heidari M.H., Alitappeh M.A. (2018). Oligoasthenoteratozoospermic (OAT) men display altered phospholipase C ζ (PLCζ) localization and a lower percentage of sperm cells expressing PLCζ and post-acrosomal sheath WW domain-binding protein (PAWP). Bosn. J. Basic Med. Sci..

[43] Rahimizadeh P., Topraggaleh T.R., Nasr-Esfahani M.H., Ziarati N., Mirshahvaladi S., Esmaeili V., Seifi S., Eftekhari-Yazdi P., Shahverdi A. (2020). The alteration of PLCζ protein expression in unexplained infertile and asthenoteratozoospermic patients: A potential effect on sperm fertilization ability. Mol. Reprod. Dev..

[44] Taylor S.L., Yoon S.Y., Morshedi M.S., Lacey D.R., Jellerette T., Fissore R.A., Oehninger S. (2010). Complete globozoospermia associated with PLCζ deficiency treated with calcium ionophore and ICSI results in pregnancy. Reprod. Biomed. Online.

[45] Heytens E., Parrington J., Coward K., Young C., Lambrecht S., Yoon S.Y., Fissore R.A., Hamer R., Deane C.M., Ruas M. (2009). Reduced amounts and abnormal forms of phospholipase C zeta (PLCζ) in spermatozoa from infertile men. Hum. Reprod..

[46] Saleh A., Kashir J., Thanassoulas A., Safieh-Garabedian B., Lai F.A., Nomikos M. (2020). Essential Role of Sperm-Specific PLC-Zeta in Egg Activation and Male Factor Infertility: An Update. Front. Cell Dev. Biol..

[47] Kashir J. (2020). Increasing associations between defects in phospholipase C zeta and conditions of male infertility: Not just ICSI failure?. J. Assist. Reprod. Genet..

[48] Heindryckx B., Van der Elst J., De Sutter P., Dhont M. (2005). Treatment option for sperm- or oocyte-related fertilization failure: Assisted oocyte activation following diagnostic heterologous ICSI. Hum. Reprod..

[49] Vanden Meerschaut F., Leybaert L., Nikiforaki D., Qian C., Heindryckx B., De Sutter P. (2013). Diagnostic and prognostic value of calcium oscillatory pattern analysis for patients with ICSI fertilization failure. Hum. Reprod..

[50] Ferrer-Buitrago M., Dhaenens L., Lu Y., Bonte D., Vanden Meerschaut F., De Sutter P., Leybaert L., Heindryckx B. (2018). Human oocyte calcium analysis predicts the response to assisted oocyte activation in patients experiencing fertilization failure after ICSI. Hum. Reprod..

[51] Meng X., Melo P., Jones C., Ross C., Mounce G., Turner K., Child T., Coward K. (2020). Use of phospholipase C zeta analysis to identify candidates for artificial oocyte activation: A case series of clinical pregnancies and a proposed algorithm for patient management. Fertil. Steril..

[52] Bonte D., Ferrer-Buitrago M., Dhaenens L., Popovic M., Thys V., De Croo I., De Gheselle S., Steyaert N., Boel A., Vanden Meerschaut F. (2019). Assisted oocyte activation significantly increases fertilization and pregnancy outcome in patients with low and total failed fertilization after intracytoplasmic sperm injection: A 17-year retrospective study. Fertil. Steril..

[53] Sun B., Yeh J. (2020). Calcium Oscillatory Patterns and Oocyte Activation During Fertilization: A Possible Mechanism for Total Fertilization Failure (TFF) in Human In Vitro Fertilization?. Reprod. Sci..

[54] Vanden Meerschaut F., Nikiforaki D., De Gheselle S., Dullaerts V., Van Den Abbeel E., Gerris J., Heindryckx B., De Sutter P. (2012). Assisted oocyte activation is not beneficial for all patients with a suspected oocyte-related activation deficiency. Hum. Reprod..

[55] Yu A., Nomikos M., Theodoridou M., Nounesis G., Lai F.A., Swann K. (2012). PLCζ causes Ca^2+^ oscillations in mouse eggs by targeting intracellular and not plasma membrane PI(4,5)P 2. Mol. Biol. Cell.

[56] Williams C.J. (2002). Signalling mechanisms of mamalian oocyte activation. Hum. Reprod. Update.

[57] Stein P., Savy V., Williams A.M., Williams C.J. (2020). Modulators of calcium signalling at fertilization: Calcium signaling at fertilization. Open Biol..

[58] Ducibella T., Huneau D., Angelichio E., Xu Z., Schultz R.M., Kopf G.S., Fissore R., Madoux S., Ozil J.-P. (2002). Egg-to-Embryo Transition Is Driven by Differential Responses to Ca^2+^ Oscillation Number. Dev. Biol..

[59] Tsaadon L., Kaplan-Kraicer R., Shalgi R. (2008). Myristoylated alanine-rich C kinase substrate, but not Ca^2+^/calmodulin-dependent protein kinase II, is the mediator in cortical granules exocytosis. Reproduction.

[60] Dupont G. (1998). Link between fertilization-induced Ca^2+^ oscillations and relief from metaphase II arrest in mammalian eggs: A model based on calmodulin-dependent kinase II activation. Biophys. Chem..

[61] (2008). Tom Ducibella and Rafael Fissore The roles of Ca^2+^, downstream protein kinases, and oscillatory signaling in regulating fertilization and the activation of development. Dev. Biol..

[62] Jones K.T. (2005). Mammalian egg activation: From Ca^2+^ spiking to cell cycle progression. Reproduction.

[63] Madgwick S., Levasseur M., Jones K.T. (2005). Calmodulin-dependent protein kinase II, and not protein kinase C, is sufficient for triggering cell-cycle resumption in mammalian eggs. J. Cell Sci..

[64] Oh J.S., Susor A., Conti M. (2011). Protein tyrosine kinase Wee1B is essential for metaphase II exit in mouse oocytes. Science.

[65] Kashir J., Deguchi R., Jones C., Coward K., Stricker S.A. (2013). Comparative biology of sperm factors and fertilization-induced calcium signals across the animal kingdom. Mol. Reprod. Dev..

[66] Nikiforaki D., Vanden Meerschaut F., Qian C., De Croo I., Lu Y., Deroo T., Van Den Abbeel E., Heindryckx B., De Sutter P. (2014). Oocyte cryopreservation and in vitro culture affect calcium signalling during human fertilization. Hum. Reprod..

[67] Tóth S., Huneau D., Banrezes B., Ozil J.P. (2006). Egg activation is the result of calcium signal summation in the mouse. Reproduction.

[68] Ozil J.P., Huneau D. (2001). Activation of rabbit oocytes: The impact of the Ca^2+^ signal regime on development. Development.

[69] Ozil J.P., Banrezes B., Tóth S., Pan H., Schultz R.M. (2006). Ca^2+^ oscillatory pattern in fertilized mouse eggs affects gene expression and development to term. Dev. Biol..

[70] Lu Y., Bonte D., Ferrer-Buitrago M., Popovic M., Neupane J., Van Der Jeught M., Leybaert L., De Sutter P., Heindryckx B. (2018). Culture conditions affect Ca^2+^ release in artificially activated mouse and human oocytes. Reprod. Fertil. Dev..

[71] Yoon S.-Y., Jellerette T., Salicioni A.M., Lee H.C., Yoo M., Coward K., Parrington J., Grow D., Cibelli J.B., Visconti P.E. (2008). Human sperm devoid of PLC, zeta 1 fail to induce Ca^2+^ release and are unable to initiate the first step of embryo development. J. Clin. Investig..

[72] Harada Y., Matsumoto T., Hirahara S., Nakashima A., Ueno S., Oda S., Miyazaki S., Iwao Y. (2007). Characterization of a sperm factor for egg activation at fertilization of the newt Cynops pyrrhogaster. Dev. Biol..

[73] Sette C., Bevilacqua A., Bianchini A., Mangia F., Geremia R., Rossi P. (1997). Parthenogenetic activation of mouse eggs by microinjection of a truncated c-kit tyrosine kinase present in spermatozoa. Development.

[74] Wu A.T.H., Sutovsky P., Manandhar G., Xu W., Katayama M., Day B.N., Park K.W., Yi Y.J., Yan W.X., Prather R.S. (2007). PAWP, a sperm-specific WW domain-binding protein, promotes meiotic resumption and pronuclear development during fertilization. J. Biol. Chem..

[75] Aarabi M., Balakier H., Bashar S., Moskovtsev S.I., Sutovsky P., Librach C.L., Oko R. (2014). Sperm-derived WW domain-binding protein, PAWP, elicits calcium oscillations and oocyte activation in humans and mice. FASEB J..

[76] Aarabi M., Balakier H., Bashar S., Moskovtsev S.I., Sutovsky P., Librach C.L., Oko R. (2014). Sperm content of postacrosomal WW binding protein is related to fertilization outcomes in patients undergoing assisted reproductive technology. Fertil. Steril..

[77] Nomikos M., Sanders J.R., Theodoridou M., Kashir J., Matthews E., Nounesis G., Lai F.A., Swann K. (2014). Sperm-specific post-acrosomal WW-domain binding protein (PAWP) does not cause Ca^2+^ release in mouse oocytes. Mol. Hum. Reprod..

[78] Nomikos M., Sanders J.R., Kashir J., Sanusi R., Buntwal L., Love D., Ashley P., Sanders D., Knaggs P., Bunkheila A. (2015). Functional disparity between human pawpand plcζ in the generation of Ca^2+^ oscillations for oocyte activation. Mol. Hum. Reprod..

[79] Satouh Y., Nozawa K., Ikawa M. (2015). Sperm postacrosomal WW domain-binding protein is not required for mouse egg activation. Biol. Reprod..

[80] Saunders C.M., Larman M.G., Parrington J., Cox L.J., Royse J., Blayney L.M., Swann K., Lai F.A. (2002). PLCζ: A sperm-specific trigger of Ca^2+^ oscillations in eggs and embryo development. Development.

[81] Rogers N.T., Hobson E., Pickering S., Lai F.A., Braude P., Swann K. (2004). Phospholipase Cζ causes Ca^2+^ oscillations and parthenogenetic activation of human oocytes. Reproduction.

[82] Yamaguchi T., Ito M., Kuroda K., Takeda S., Tanaka A. (2017). Cell Calcium The establishment of appropriate methods for egg-activation by human PLCZ1 RNA injection into human oocyte. Cell Calcium.

[83] Yoon S., Eum J.H., Lee J.E., Lee H.C., Kim Y.S., Han J.E., Won H.J., Park S.H., Shim S.H., Lee W.S. (2012). Recombinant human phospholipase C zeta 1 induces intracellular calcium oscillations and oocyte activation in mouse and human oocytes. Hum. Reprod..

[84] Theodoridou M., Nomikos M., Parthimos D., Gonzalez-Garcia J.R., Elgmati K., Calver B.L., Sideratou Z., Nounesis G., Swann K., Lai F.A. (2013). Chimeras of sperm PLCζ reveal disparate protein domain functions in the generation of intracellular Ca^2+^ oscillations in mammalian eggs at fertilization. Mol. Hum. Reprod..

[85] Knott J.G., Kurokawa M., Fissore R.A., Schultz R.M., Williams C.J. (2005). Transgenic RNA interference reveals role for mouse sperm phospholipase Cζ in triggering Ca^2+^ oscillations during fertilization. Biol. Reprod..

[86] Escoffier J., Lee H.C., Yassine S., Zouari R., Martinez G., Karaouzène T., Coutton C., Kherraf Z.E., Halouani L., Triki C. (2016). Homozygous mutation of PLCZ1 leads to defective human oocyte activation and infertility that is not rescued by the WW-binding protein PAWP. Hum. Mol. Genet..

[87] Hachem A., Godwin J., Ruas M., Lee H.C., Buitrago M.F., Ardestani G., Bassett A., Fox S., Navarrete F., De Sutter P. (2017). Plcζ is the physiological trigger of the Ca^2+^ oscillations that induce embryogenesis in mammals but conception can occur in its absence. Development.

[88] Nozawa K., Satouh Y., Fujimoto T., Oji A., Ikawa M. (2018). Sperm-borne phospholipase C zeta-1 ensures monospermic fertilization in mice. Sci. Rep..

[89] Sato K.I., Tokmakov A.A., Iwasaki T., Fukami Y. (2000). Tyrosine kinase-dependent activation of phospholipase Cγ is required for calcium transient in Xenopus egg fertilization. Dev. Biol..

[90] Runft L.L., Carroll D.J., Gillett J., Giusti A.F., O’Neill F.J., Foltz K.R. (2004). Identification of a starfish egg PLC-γ that regulates Ca^2+^ release at fertilization. Dev. Biol..

[91] Coward K., Owen H., Tunwell R., Swann K., Parrington J. (2007). Phospholipid binding properties and functional characterization of a sea urchin phospholipase Cδ in urchin and mouse eggs. Biochem. Biophys. Res. Commun..

[92] Yin X., Eckberg W.R. (2009). Characterization of phospholipases C ß and γ and their possible roles in chaetopterus egg activation. Mol. Reprod. Dev..

[93] Igarashi H., Knott J.G., Schultz R.M., Williams C.J. (2007). Alterations of PLCβ1 in mouse eggs change calcium oscillatory behavior following fertilization. Dev. Biol..

[94] Rybouchkin A., Dozortsev D., De Sutter P., Qian C., Dhont M. (1995). Andrology: Intracytoplasmic injection of human spermatozoa into mouse oocytes: A useful model to investigate the oocyte-activating capacity and the karyotype of human spermatozoa. Hum. Reprod..

[95] Goud P.T., Goud A.P., Rybouchkin A.V., De Sutter P., Dhont M. (1998). Chromatin decondensation, pronucleus formation, metaphase entry and chromosome complements of human spermatozoa after intracytoplasmic sperm injection into hamster oocytes. Hum. Reprod..

[96] Jones E.L., Mudrak O., Zalensky A.O. (2010). Kinetics of human male pronuclear development in a heterologous ICSI model. J. Assist. Reprod. Genet..

[97] Ahmadi A., Bongso A., Ng S.C. (1996). Intracytoplasmic injection of human sperm into the hamster oocyte (Hamster ICSI Assay) as a test for fertilizing capacity of the severe male-factor sperm. J. Assist. Reprod. Genet..

[98] Chi H.J., Koo J.J., Song S.J., Lee J.Y., Chang S.S. (2004). Successful fertilization and pregnancy after intracytoplasmic sperm injection and oocyte activation with calcium ionophore in a normozoospermic patient with extremely low fertilization rates in intracytoplasmic sperm injection cycles. Fertil. Steril..

[99] Schatten H., Sun Q.Y. (2009). The role of centrosomes in mammalian fertilization and its significance for ICSI. Mol. Hum. Reprod..

[100] Avidor-Reiss T., Khire A., Fishman E.L., Jo K.H. (2015). Atypical centrioles during sexual reproduction. Front. Cell Dev. Biol..

[101] Sun Q.Y., Schatten H. (2007). Centrosome inheritance after fertilization and nuclear transfer in mammals. Adv. Exp. Med. Biol..

[102] Terada Y., Nakamura S.I., Simerly C., Hewitson L., Murakami T., Yaegashi N., Okamura K., Schatten G. (2004). Centrosomal Function Assessment in Human Sperm Using Heterologous ICSI with Rabbit Eggs: A New Male Factor Infertility Assay. Mol. Reprod. Dev..

[103] Yoshimoto-Kakoi T., Terada Y., Tachibana M., Murakami T., Yaegashi N., Okamura K. (2008). Assessing centrosomal function of infertile males using heterologous ICSI. Syst. Biol. Reprod. Med..

[104] Nakamura S.I., Terada Y., Horiuchi T., Emuta C., Murakami T., Yaegashi N., Okamura K. (2001). Human sperm aster formation and pronuclear decondensation in bovine eggs following intracytoplasmic sperm injection using a piezo-driven pipette: A novel assay for human sperm centrosomal function. Biol. Reprod..

[105] Nakamura S., Terada Y., Horiuchi T., Emuta C., Murakami T., Yaegashi N., Okamura K. (2002). Analysis of the human sperm centrosomal function and the oocyte activation ability in a case of globozoospermia, by ICSI into bovine oocytes. Hum. Reprod..

[106] Rawe V.Y., Terada Y., Nakamura S., Chillik C.F., Olmedo S.B., Chemes H.E. (2002). A pathology of the sperm centriole responsible for defective sperm aster formation, syngamy and cleavage. Hum. Reprod..

[107] Heindryckx B., De Gheselle S., Gerris J., Dhont M., De Sutter P. (2008). Efficiency of assisted oocyte activation as a solution for failed intracytoplasmic sperm injection. Reprod. Biomed. Online.

[108] Rybouchkin A.V., De Sutter P., Van Der Straeten F., Dhont M., Quatacker J. (1997). Fertilization and pregnancy after assisted oocyte activation and intracytoplasmic sperm injection in a case of round-headed sperm associated with deficient oocyte activation capacity. Fertil. Steril..

[109] Heytens E., Schmitt-John T., Moser J.M., Jensen N.M., Soleimani R., Young C., Coward K., Parrington J., De Sutter P. (2010). Reduced fertilization after ICSI and abnormal phospholipase C zeta presence in spermatozoa from the wobbler mouse. Reprod. Biomed. Online.

[110] Tesarik J., Rienzi L., Ubaldi F., Mendoza C., Greco E. (2002). Use of a modified intracytoplasmic sperm injection technique to overcome sperm-borne and oocyte-borne oocyte activation failures. Fertil. Steril..

[111] Araki Y., Yoshizawa M., Abe H., Murase Y., Araki Y. (2004). Use of mouse oocytes to evaluate the ability of human sperm to activate oocytes after failure of activation by intracytoplasmic sperm injection. Zygote.

[112] Egashira A., Murakami M., Haigo K., Horiuchi T., Kuramoto T. (2009). A successful pregnancy and live birth after intracytoplasmic sperm injection with globozoospermic sperm and electrical oocyte activation. Fertil. Steril..

[113] Kyono K., Nakajo Y., Nishinaka C., Hattori H., Kyoya T., Ishikawa T., Abe H., Araki Y. (2009). A birth from the transfer of a single vitrified-warmed blastocyst using intracytoplasmic sperm injection with calcium ionophore oocyte activation in a globozoospermic patient. Fertil. Steril..

[114] Bonte D., Thys V., De Sutter P., Boel A., Leybaert L., Heindryckx B. (2020). Vitrification negatively affects the Ca^2+^-releasing and activation potential of mouse oocytes, but vitrified oocytes are potentially useful for diagnostic purposes. Reprod. Biomed. Online.

[115] Morozumi K., Shikano T., Miyazaki S., Yanagimachi R. (2006). Simultaneous removal of sperm plasma membrane and acrosome before intracytoplasmic sperm injection improves oocyte activation/embryonic development. Proc. Natl. Acad. Sci. USA.

[116] Cox L.J., Larman M.G., Saunders C.M., Hashimoto K., Swann K., Lai F.A. (2002). Sperm phospholipase Cζ from humans and cynomolgus monkeys triggers Ca^2+^ oscillations, activation and development of mouse oocytes. Reproduction.

[117] Nomikos M., Theodoridou M., Elgmati K., Parthimos D., Calver B.L., Buntwal L., Nounesis G., Swann K., Anthony Lai F. (2014). Human PLCζ exhibits superior fertilization potency over mouse PLCζ in triggering the Ca^2+^ oscillations required for mammalian oocyte activation. Mol. Hum. Reprod..

[118] Nikiforaki D., Vanden Meerschaut F., De Gheselle S., Qian C., Van Den Abbeel E., De Vos W.H., Deroo T., De Sutter P., Heindryckx B. (2014). Sperm involved in recurrent partial hydatidiform moles cannot induce the normal pattern of calcium oscillations. Fertil. Steril..

[119] Dai J., Zheng W., Dai C., Guo J., Lu C., Gong F., Li Y., Zhou Q., Lu G., Lin G. (2019). New biallelic mutations in WEE2: Expanding the spectrum of mutations that cause fertilization failure or poor fertilization. Fertil. Steril..

[120] Busardò F.P., Gulino M., Napoletano S., Zaami S., Frati P. (2014). The Evolution of Legislation in the Field of Medically Assisted Reproduction and Embryo Stem Cell Research in European Union Members. Biomed. Res. Int..

[121] De Gheselle N., De Croo V., Heindryckx L., Van den Abbeel D.S. The effect of smooth endoplasmic reticulum aggregates in human oocytes on calcium signalling and the significance for oocyte collection cycle. Proceedings of the Abstracts of the 30th Annual Meeting of ESHRE.

[122] Leemans B., Gadella B.M., Stout T.A.E., Heras S., Smits K., Ferrer-Buitrago M., Claes E., Heindryckx B., De Vos W.H., Nelis H. (2015). Procaine induces cytokinesis in horse oocytes via a pH-dependent mechanism. Biol. Reprod..

[123] Ajduk A., Ilozue T., Windsor S., Yu Y., Seres K.B., Bomphrey R.J., Tom B.D., Swann K., Thomas A., Graham C. (2011). Rhythmic actomyosin-driven contractions induced by sperm entry predict mammalian embryo viability. Nat. Commun..

[124] Swann K., Windsor S., Campbell K., Elgmati K., Nomikos M., Zernicka-Goetz M., Amso N., Lai F.A., Thomas A., Graham C. (2012). Phospholipase C-ζ-induced Ca^2+^ oscillations cause coincident cytoplasmic movements in human oocytes that failed to fertilize after intracytoplasmic sperm injection. Fertil. Steril..

[125] Milewski R., Szpila M., Ajduk A. (2018). Dynamics of cytoplasm and cleavage divisions correlates with preimplantation embryo development. Reproduction.

[126] Ramadan W.M., Kashir J., Jones C., Coward K. (2012). Oocyte activation and phospholipase C zeta (PLCζ): Diagnostic and therapeutic implications for assisted reproductive technology. Cell Commun. Signal..

[127] Amdani S.N., Yeste M., Jones C., Coward K. (2016). Phospholipase C zeta (PLCζ) and male infertility: Clinical update and topical developments. Adv. Biol. Regul..

[128] Nomikos M., Kashir J., Swann K., Lai F.A. (2013). Sperm PLCζ: From structure to Ca^2+^ oscillations, egg activation and therapeutic potential. FEBS Lett..

[129] Nomikos M., Elgmati K., Theodoridou M., Georgilis A., Gonzalez-Garcia J.R., Nounesis G., Swann K., Lai F.A. (2011). Novel regulation of PLCζ activity via its XY-linker. Biochem. J..

[130] Mu J., Zhang Z., Wu L., Fu J., Chen B., Yan Z., Li B., Zhou Z., Wang W., Zhao L. (2020). The identification of novel mutations in PLCZ1 responsible for human fertilization failure and a therapeutic intervention by artificial oocyte activation. Mol. Hum. Reprod..

[131] Yuan P., Yang C., Ren Y., Yan J., Nie Y., Yan L., Qiao J. (2020). A novel homozygous mutation of phospholipase C zeta leading to defective human oocyte activation and fertilization failure. Hum. Reprod..

[132] Kleene K.C. (2013). Connecting cis-elements and trans-factors with mechanisms of developmental regulation of mRNA translation in meiotic and haploid mammalian spermatogenic cells. Reproduction.

[133] Dai J., Dai C., Guo J., Zheng W., Zhang T., Li Y., Lu C., Gong F., Lu G., Lin G. (2020). Novel homozygous variations in PLCZ1 lead to poor or failed fertilization characterized by abnormal localization patterns of PLCζ in sperm. Clin. Genet..

[134] Wu L., Chen H., Li D., Song D., Chen B., Yan Z., lyu Q., Wang L., Kuang Y., Li B. (2019). Novel mutations in PATL2: Expanding the mutational spectrum and corresponding phenotypic variability associated with female infertility. J. Hum. Genet..

[135] Chen B., Li B., Li D., Yan Z., Mao X., Xu Y., Mu J., Li Q., Jin L., He L. (2017). Novel mutations and structural deletions in TUBB8: Expanding mutational and phenotypic spectrum of patients with arrest in oocyte maturation, fertilization or early embryonic development. Hum. Reprod..

[136] Alazami A.M., Awad S.M., Coskun S., Al-Hassan S., Hijazi H., Abdulwahab F.M., Poizat C., Alkuraya F.S. (2015). TLE6 mutation causes the earliest known human embryonic lethality. Genome Biol..

[137] Aghajanpour S., Ghaedi K., Salamian A., Deemeh M.R., Tavalaee M., Moshtaghian J., Parrington J., Nasr-Esfahani M.H. (2011). Quantitative expression of phospholipase C zeta, as an index to assess fertilization potential of a semen sample. Hum. Reprod..

[138] Tavalaee M., Nasr-Esfahani M.H. (2016). Expression profile of PLCζ, PAWP, and TR-KIT in association with fertilization potential, embryo development, and pregnancy outcomes in globozoospermic candidates for intra-cytoplasmic sperm injection and artificial oocyte activation. Andrology.

[139] Wang F., Zhang J., Kong S., Li C., Zhang Z., He X., Wu H., Tang D., Zha X., Tan Q. (2020). A homozygous nonsense mutation of PLCZ1 cause male infertility with oocyte activation deficiency. J. Assist. Reprod. Genet..

[140] Kashir J., Jones C., Lee H.C., Rietdorf K., Nikiforaki D., Durrans C., Ruas M., Tee S.T., Heindryckx B., Galione A. (2011). Loss of activity mutations in phospholipase C zeta ( PLC z ) abolishes calcium oscillatory ability of human recombinant protein in mouse oocytes. Hum. Reprod..

[141] Nazarian H., Azad N., Nazari L., Piryaei A., Heidari M.H., Masteri-Farahani R., Karimi M., Ghaffari-Novin M. (2019). Effect of artificial oocyte activation on intra-cytoplasmic sperm injection outcomes in patients with lower percentage of sperm containing phospholipase Cζ: A randomized clinical trial. J. Reprod. Infertil..

[142] Yelumalai S., Yeste M., Jones C., Amdani S.N., Kashir J., Mounce G., Da Silva S.J.M., Barratt C.L., McVeigh E., Coward K. (2015). Total levels, localization patterns, and proportions of sperm exhibiting phospholipase C zeta are significantly correlated with fertilization rates after intracytoplasmic sperm injection. Fertil. Steril..

[143] Grasa P., Coward K., Young C., Parrington J. (2008). The pattern of localization of the putative oocyte activation factor, phospholipase Cζ, in uncapacitated, capacitated, and ionophore-treated human spermatozoa. Hum. Reprod..

[144] Escoffier J., Yassine S., Lee H.C., Martinez G., Delaroche J., Coutton C., Karaouzéne T., Zouari R., Metzler-Guillemain C., Pernet-Gallay K. (2014). Subcellular localization of phospholipase Cz in human sperm and its absence in DPY19L2-deficient sperm are consistent with its role in oocyte activation. Mol. Hum. Reprod..

[145] Ferrer-Vaquer A., Barragan M., Freour T., Vernaeve V., Vassena R. (2016). PLCζ sequence, protein levels, and distribution in human sperm do not correlate with semen characteristics and fertilization rates after ICSI. J. Assist. Reprod. Genet..

[146] Kashir J., Jones C., Mounce G., Ramadan W.M., Lemmon B., Heindryckx B., De Sutter P., Parrington J., Turner K., Child T. (2013). Variance in total levels of phospholipase C zeta (PLC-ζ) in human sperm may limit the applicability of quantitative immunofluorescent analysis as a diagnostic indicator of oocyte activation capability. Fertil. Steril..

[147] Kashir J., Buntwal L., Nomikos M., Calver B.L., Stamatiadis P., Ashley P., Vassilakopoulou V., Sanders D., Knaggs P., Livaniou E. (2017). Antigen unmasking enhances visualization efficacy of the oocyte activation factor, phospholipase C zeta, in mammalian sperm. Mol. Hum. Reprod..

[148] Cheung S., Xie P., Parrella A., Keating D., Rosenwaks Z., Palermo G.D. (2020). Identification and treatment of men with phospholipase Cζ–defective spermatozoa. Fertil. Steril..

[149] Ferrer-Buitrago M., Bonte D., De Sutter P., Leybaert L., Heindryckx B. (2018). Single Ca^2+^ transients vs oscillatory Ca^2+^ signaling for assisted oocyte activation: Limitations and benefits. Reproduction.

[150] Yanagida K., Katayose H., Yazawa H., Kimura Y., Sato A., Yanagimachi H., Yanagimachi R. (1999). Successful fertilization and pregnancy following ICSI and electrical oocyte activation. Hum. Reprod..

[151] Mansour R., Fahmy I., Tawab N.A., Kamal A., El-Demery Y., Aboulghar M., Serour G. (2009). Electrical activation of oocytes after intracytoplasmic sperm injection: A controlled randomized study. Fertil. Steril..

[152] Baltaci V., Ayvaz Ö.Ü., Ünsal E., Akta Y., Baltac A., Turhan F., Özcan S., Sönmezer M. (2010). The effectiveness of intracytoplasmic sperm injection combined with piezoelectric stimulation in infertile couples with total fertilization failure. Fertil. Steril..

[153] Ebner T., Moser M., Sommergruber M., Jesacher K., Tews G. (2004). Complete oocyte activation failure after ICSI can be overcome by a modified injection technique. Hum. Reprod..

[154] Ebner T., Montag M., Montag M., Van Der Ven K., Van Der Ven H., Ebner T., Shebl O., Oppelt P., Hirchenhain J., Krüssel J. (2015). Live birth after artificial oocyte activation using a ready-to-use ionophore: A prospective multicentre study. Reprod. Biomed. Online.

[155] Montag M., Köster M., Van Der Ven K., Bohlen U., Van Der Ven H. (2012). The benefit of artificial oocyte activation is dependent on the fertilization rate in a previous treatment cycle. Reprod. Biomed. Online.

[156] Tejera A., Mollá M., Muriel L., Remohí J., Pellicer A., De Pablo J.L. (2008). Successful pregnancy and childbirth after intracytoplasmic sperm injection with calcium ionophore oocyte activation in a globozoospermic patient. Fertil. Steril..

[157] Nasr-Esfahani M.H., Razavi S., Javdan Z., Tavalaee M. (2008). Artificial oocyte activation in severe teratozoospermia undergoing intracytoplasmic sperm injection. Fertil. Steril..

[158] Li J., Zheng X., Lian Y., Li M., Lin S., Zhuang X., Chen L., Liu P., Qiao J. (2019). Artificial oocyte activation improves cycles with prospects of ICSI fertilization failure: A sibling oocyte control study. Reprod. Biomed. Online.

[159] Nikiforaki D., Vanden Meerschaut F., De Roo C., Lu Y., Ferrer-Buitrago M., De Sutter P., Heindryckx B. (2016). Effect of two assisted oocyte activation protocols used to overcome fertilization failure on the activation potential and calcium releasing pattern. Fertil. Steril..

[160] Versieren K., Heindryckx B., Lierman S., Gerris J., De Sutter P. (2010). Developmental competence of parthenogenetic mouse and human embryos after chemical or electrical activation. Reprod. Biomed. Online.

[161] Vanden Meerschaut F., Nikiforaki D., De Roo C., Lierman S., Qian C., Schmitt-John T., De Sutter P., Heindryckx B. (2013). Comparison of pre-and post-implantation development following the application of three artificial activating stimuli in a mouse model with round-headed sperm cells deficient for oocyte activation. Hum. Reprod..

[162] Carvacho I., Lee H.C., Fissore R.A., Clapham D.E. (2014). TRPV3 mediates Sr influx during mouse egg activation. Cell Rep..

[163] Lu Y., Reddy R., Ferrer Buitrago M., Vander Jeught M., Neupane J., De Vos W.H., Van den Abbeel E., Lierman S., De Sutter P., Heindryckx B. (2018). Strontium fails to induce Ca^2+^ release and activation in human oocytes despite the presence of functional TRPV3 channels. Hum. Reprod. Open.

[164] Yanagida K., Morozumi K., Katayose H., Hayashi S., Sato A. (2006). Successful pregnancy after ICSI with strontium oocyte activation in low rates of fertilization. Reprod. Biomed. Online.

[165] Kim J.W., Kim S.D., Yang S.H., Yoon S.H., Jung J.H., Lim J.H. (2014). Successful pregnancy after SrCl2 oocyte activation in couples with repeated low fertilization rates following calcium ionophore treatment. Syst. Biol. Reprod. Med..

[166] Fawzy M., Emad M., Mahran A., Sabry M., Fetih A.N., Abdelghafar H., Rasheed S. (2018). Artificial oocyte activation with SrCl_2_ or calcimycin after ICSI improves clinical and embryological outcomes compared with ICSI alone: Results of a randomized clinical trial. Hum. Reprod..

[167] Norozi-Hafshejani M., Tavalaee M., Azadi L., Bahadorani M., Nasr-Esfahani M.H. (2018). Effects of assisted oocyte activation with calcium-ionophore and strontium chloride on in vitro ICSI outcomes. Iran. J. Basic Med. Sci..

[168] Bernhardt M.L., Kong B.Y., Kim A.M., O’Halloran T.V., Woodruff T.K. (2012). A Zinc-Dependent Mechanism Regulates Meiotic Progression in Mammalian Oocytes. Biol. Reprod..

[169] Duncan F.E., Que E.L., Zhang N., Feinberg E.C., O’Halloran T.V., Woodruff T.K. (2016). The zinc spark is an inorganic signature of human egg activation. Sci. Rep..

[170] De Sutter P., Dozortsev D., Cieslak J., Wolf G., Verlinsky Y., Dyban A. (1992). Parthenogenetic activation of human oocytes by puromycin. J. Assist. Reprod. Genet..

[171] Rogers N.T., Halet G., Piao Y., Carroll J., Ko M.S.H., Swann K. (2006). The absence of a Ca^2+^ signal during mouse egg activation can affect parthenogenetic preimplantation development, gene expression patterns, and blastocyst quality. Reproduction.

[172] Phillips K.P., Petrunewich M.A.F., Collins J.L., Booth R.A., Liu X.J., Baltz J.M. (2002). Inhibition of MEK or cdc2 kinase parthenogenetically activates mouse eggs and yields the same phenotypes as Mos-/-parthenogenotes. Dev. Biol..

[173] Nomikos M., Ph D., Yu Y., Ph D., Elgmati K., Sc M., Theodoridou M., Sc M. (2013). Phospholipase C z rescues failed oocyte activation in a prototype of male factor infertility. Fertil. Steril..

[174] Ferrer-Buitrago M., Tilleman L., Thys V., Hachem A., Boel A., Van Nieuwerburgh F., Deforce D., Leybaert L., De Sutter P., Parrington J. (2020). Comparative study of pre-implantation development following distinct assisted oocyte activation protocols in a PLC-Zeta knockout mouse model Running. Mol. Hum. Reprod..

[175] Van Blerkom J., Cohen J., Johnson M. (2015). A plea for caution and more research in the “experimental” use of ionophores in ICSI. Reprod. Biomed. Online.

[176] Vanden Meerschaut F., D’Haeseleer E., Gysels H., Thienpont Y., Dewitte G., Heindryckx B., Oostra A., Roeyers H., Van Lierde K., De Sutter P. (2014). Neonatal and neurodevelopmental outcome of children aged 3-10 years born following assisted oocyte activation. Reprod. Biomed. Online.

[177] Deemeh M.R., Tavalaee M., Nasr-Esfahani M.H. (2015). Health of children born through artificial oocyte activation: A pilot study. Reprod. Sci..

[178] Mateizel I., Verheyen G., Van de Velde H., Tournaye H., Belva F. (2018). Obstetric and neonatal outcome following ICSI with assisted oocyte activation by calcium ionophore treatment. J. Assist. Reprod. Genet..

[179] Li B., Zhou Y., Yan Z., Li M., Xue S., Cai R., Fu Y., Hong Q., Long H., Yin M. (2019). Pregnancy and neonatal outcomes of artificial oocyte activation in patients undergoing frozen–thawed embryo transfer: A 6-year population-based retrospective study. Arch. Gynecol. Obstet..

[180] Takisawa T., Sato Y., Tasaka A., Ito Y., Nakamura Y., Hattori H. (2011). Effect of oocyte activation by calcium ionophore A23187 or strontium chloride in patients with low fertilization rates and follow-up of babies. Fertil. Steril..

[181] Kyono K., Kumagai S., Nishinaka C., Nakajo Y., Uto H., Toya M., Sugawara J., Araki Y. (2008). Birth and follow-up of babies born following ICSI using SrCl2 oocyte activation. Reprod. Biomed. Online.

[182] Capalbo A., Ottolini C.S., Griffin D.K., Ubaldi F.M., Handyside A.H., Rienzi L. (2016). Artificial oocyte activation with calcium ionophore does not cause a widespread increase in chromosome segregation errors in the second meiotic division of the oocyte. Fertil. Steril..

[183] Ferrer-Buitrago M., Bonte D., Dhaenens L., Vermorgen S., Lu Y., De Sutter P., Heindryckx B. (2019). Assessment of the calcium releasing machinery in oocytes that failed to fertilize after conventional ICSI and assisted oocyte activation. Reprod. Biomed. Online.

[184] Yang X., Shu L., Cai L., Sun X., Cui Y., Liu J. (2019). Homozygous missense mutation Arg207Cys in the WEE2 gene causes female infertility and fertilization failure. J. Assist. Reprod. Genet..

[185] Ebner T., Oppelt P., Wober M., Staples P., Mayer R.B., Sonnleitner U., Bulfon-Vogl S., Gruber I., Haid A.E., Shebl O. (2015). Treatment with Ca^2+^ ionophore improves embryo development and outcome in cases with previous developmental problems: A prospective multicenter study. Hum. Reprod..

[186] Lv M., Zhang D., He X., Chen B., Li Q., Ding D., Hao Y., Xue R., Ji D., Zou W. (2020). Artificial oocyte activation to improve reproductive outcomes in couples with various causes of infertility: A retrospective cohort study. Reprod. Biomed. Online.

[187] Kashir J., Konstantinidis M., Jones C., Lemmon B., Chang Lee H., Hamer R., Heindryckx B., Deane C.M., De Sutter P., Fissore R.A. (2012). A maternally inherited autosomal point mutation in human phospholipase C zeta (PLCζ) leads to male infertility. Hum. Reprod..

